# Sperm-Leucylaminopeptidases are required for male fertility as structural components of mitochondrial paracrystalline material in *Drosophila melanogaster* sperm

**DOI:** 10.1371/journal.pgen.1007987

**Published:** 2019-02-25

**Authors:** Barbara Laurinyecz, Viktor Vedelek, Attila L. Kovács, Kinga Szilasi, Zoltán Lipinszki, Csilla Slezák, Zsuzsanna Darula, Gábor Juhász, Rita Sinka

**Affiliations:** 1 Department of Genetics, University of Szeged, Szeged, Hungary; 2 Department of Anatomy, Eötvös Loránd University, Budapest, Hungary; 3 Institute of Biochemistry and MTA SZBK Lendület Laboratory of Cell Cycle Regulation, Biological Research Centre, Hungarian Academy of Sciences, Szeged, Hungary; 4 Laboratory of Proteomics Research, Biological Research Centre, Hungarian Academy of Sciences, Szeged, Hungary; College de France CNRS, FRANCE

## Abstract

*Drosophila melanogaster* sperm reach an extraordinary long size, 1.8 mm, by the end of spermatogenesis. The mitochondrial derivatives run along the entire flagellum and provide structural rigidity for flagellar movement, but its precise function and organization is incompletely understood. The two mitochondrial derivatives differentiate and by the end of spermatogenesis the minor one reduces its size and the major one accumulates paracrystalline material inside it. The molecular constituents and precise function of the paracrystalline material have not yet been revealed. Here we purified the paracrystalline material from mature sperm and identified by mass spectrometry Sperm-Leucylaminopeptidase (S-Lap) family members as important constituents of it. To study the function of S-Lap proteins we show the characterization of classical mutants and RNAi lines affecting of the *S-Lap* genes and the analysis of their mutant phenotypes. We show that the male sterile phenotype of the S-Lap mutants is caused by defects in paracrystalline material accumulation and abnormal structure of the elongated major mitochondrial derivatives. Our work shows that S-Lap proteins localize and accumulate in the paracrystalline material of the major mitochondrial derivative. Therefore, we propose that S-Lap proteins are important constituents of the paracrystalline material of *Drosophila melanogaster* sperm.

## Author summary

Mitochondria are essential organelles of developing spermatids, but many of the factors that necessary to mitochondrial differentiation, elongation and structural composition during spermatogenesis need to be identified. In almost all insect species there are two mitochondrial derivatives in mature sperm. One or both mitochondrial derivatives accumulate paracrystalline material; however, its exact molecular composition and function are still unresolved. We purified paracrystalline material from *Drosophila melanogaster* sperm and identified Sperm-Leucylaminopeptidase (S-Lap) family members as a major constituent of it. We characterized mutant alleles of *S-Lap* genes and showed that their male sterile phenotype is caused by defects in paracrystalline material accumulation and abnormal structure of the elongated major mitochondrial derivatives. In this study we provide the first experimental proof that S-Lap proteins are important structural constituents of the paracrystalline material of the *Drosophila melanogaster’s* sperm. Our results could contribute to the better understanding of the formation of special ordered protein structures, like paracrystalline material, and filamentous enzymes.

## Introduction

Insects are the record-holders for the sperm size; they have the smallest and the biggest flagellated sperm among the animals. Insect sperm contain the same structural elements as mammalian sperm, such as the plasma membrane, the acrosome, the elongated nucleus, the axoneme and the mitochondria. The length of sperm among insects varies extremely, between 7 μm and nearly 6 cm [1]. The longest sperm can be found in the Drosophila genus with 58.29 mm in *D*. *bifurca*, 24.29 mm in *D*. *kanekoi* or 23.3 mm in *D*. *hydei* [2–3]. The genetic model *Drosophila melanogaster* also has giant sperm with a length of 1.8 mm. The normal width of insect sperm is between 0.5-0.7 μm, but in many cases, sperm exceed the average diameter, usually because of the extremely large mitochondrial derivatives, which run along the entire flagellum. The size ratio between the axoneme (0.23 μm in every case) and the mitochondria is generally 1:2 or 1:3. The widest sperm described so far belongs to *Zorotypus impolitus* with a 1:13 axoneme:mitochondria size ratio [4]. The mitochondrial derivatives fill most of the volume of the mature sperm in insects, and the process of mitochondrial development follows the same basic steps (reviewed in [5]). During early stages of spermatogenesis the mitochondria are dispersed in the cytoplasm, then by the end of meiosis, they aggregate and fuse into a two-part spherical mass, called the nebenkern. The two mitochondrial derivatives elongate along the entire length of the mature sperm tail and stay in close association with the axoneme. Interestingly, in almost all studied insect species one or both mitochondrial derivatives accumulate paracrystalline material; however, its exact molecular composition and function are still unresolved. There are several hypotheses about the function of the paracrystalline material in insect spermatids. Tokuyasu suggested that paracrystalline material gives an elastic feature to the sperm tail and contributes to sperm undulation by the storage or release of kinetic energy [6]. In almost all examined insects, the complete sperm enters the egg at fertilization and degraded in the first stages of the embryonic development; however, it is not clear whether the paracrystalline material of the mitochondrial derivatives has a role in fertilization or after it [6–7]. There are electron microscopic observations of the structure of the sperm mitochondrial paracrystalline material from *D*. *hydei* and *D*. *melanogaster*, wherein a periodic pattern is observed with 300 Å and 260 Å periodicity in the longitudinal sections [8]. In cross sections of mature *D*. *melanogaster* sperm, the hexagonal arrangements of the paracrystalline material structure are clearly visible [7]. So far the most targeted experiments on the molecular composition of mitochondrial paracrystalline material were conducted on the sperm of *Notonecta glauca* (backswimmer). The electron microscopic studies of the backswimmer sperm showed paracrystalline material crystals in 45 nm periodicity with 20 nm subperiods, which is determined by the coiling of 2 nm thick filaments made up from globular components. They also isolated the paracrystalline material biochemically and found that the proteins in the SDS-resistant fraction of isolated sperm are in the range of 50-55 kDa molecular weight [9].

The proteome analyses by mass spectrometry provided detailed data about the protein components of *D*. *melanogaster* testis and sperm [10, 11, 12]. Based on the fact that mitochondria comprise almost 50% of the mature sperm and the major mitochondrial derivative is packed with paracrystalline material, the protein components of the paracrystalline material are expected to be an abundant constituent of the sperm proteome. The major elements found in the *Drosophila* sperm proteome are members of the Sperm-Leucylaminopeptidase (S-Lap) protein family, which consists of eight proteins [12]. All members of the S-Lap family show testis-specific expression [13, 14, 15]. Very interestingly, Dorus *et al*. made the evolutionary characterization of the expanded gene family and proposed that the conserved amino acid changes in the divalent cation binding and catalytic residues suggest the neofunctionalization of the S-Lap proteins in sperm [16].

In this work we isolated the *D*. *melanogaster* paracrystalline material from sperm and performed a proteomic analysis that identified all S-Lap protein family members as components of the crystal. We have characterized the mutant alleles of the S-Lap gene family and found that mutation even in a single S-Lap gene resulted in male sterility. We demonstrated that the mitochondrial abnormalities in the elongated spermatids are responsible for the sterile phenotype of the S-Lap mutants. We localized the S-Lap proteins to the paracrystalline material of elongated major mitochondrial derivatives with immunogold labeling. Our results strongly indicate that S-Lap proteins are the crucial components of the paracrystalline material of the major mitochondrial derivative of *D*. *melanogaster* sperm.

## Results

### Purification of the paracrystalline material

Despite the general occurrence of paracrystalline material in mitochondrial derivatives of insect sperm, there are no data about its molecular components. Baccetti *et al*. isolated the paracrystalline material of mitochondrial derivatives in a cross-section or extract of testes from adult backswimmer *Notonecta glauca* using a treatment of samples with an appropriate concentration of the anionic detergent, SDS. This extraction does not solubilize the paracrystalline material, only the axoneme and the membranes are dissolved, therefore it could be easily separated from the non-paracrystalline part of the sperm by centrifugation. The isolated paracrystalline was then fractionated on SDS-PAGE and the protein run around 50-55 kDa was called crystallomitin. However, the molecular identity and function of crystallomitin remained unresolved [9].

**Fig 1:**
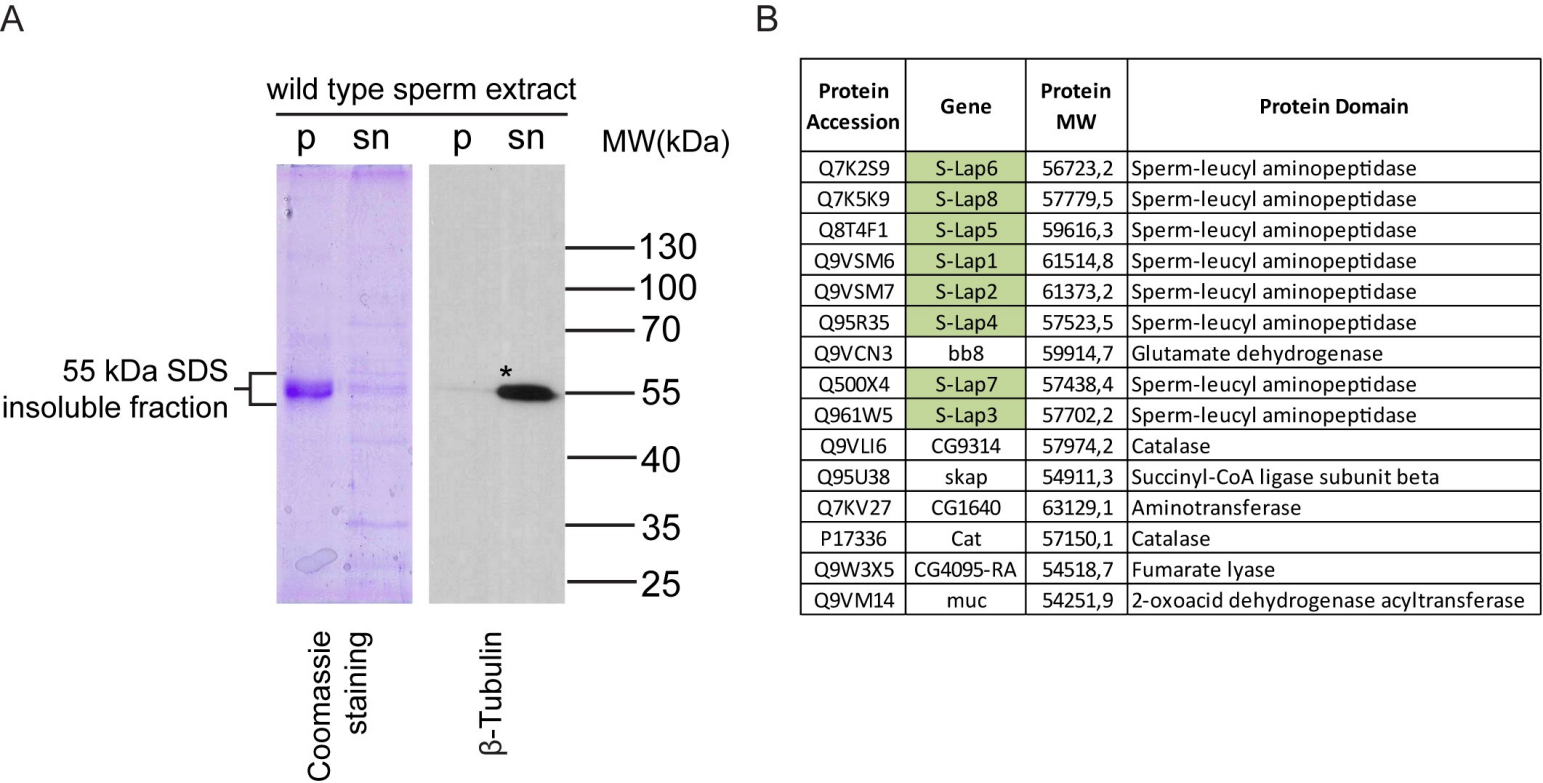
Strong abundance of proteins in the insoluble pellet of wild-type sperm extract. (A) Coomassie Brilliant Blue stained gel and immunoblot analysis of the SDS-soluble supernatant (sn) and the SDS-resistant pellet (p) fractions isolated from wild-type sperm lysates by SDS extraction. *β-Tubulin is mainly present in the supernatant fraction of the lysate. (B) List of proteins identified only in the 50-60 kDa fraction of SDS-resistant pellet of sperm extract. Green highlights represent the Sperm-Leucylaminopeptidases.

In order to identify the molecular composition of the paracrystalline material in *Drosophila melanogaster*, we isolated the SDS-resistant fraction of *Drosophila melanogaster* wild-type sperm. We separated the soluble supernatant and the SDS-resistant insoluble pellet by centrifugation and observed a strong abundance of proteins around 55 kDa in the insoluble pellet fraction on the Coomassie Brilliant Blue stained denaturing polyacrylamide gel (Fig 1A).

Based on the data from backswimmer, we assumed that this pellet and the proteins in the ~55 kDa region contain components of the paracrystalline material. We compared the protein composition of the ~55 kDa insoluble and the soluble fraction of wild-type *Drosophila melanogaster* sperm by proteomic analysis. To identify the components of the pellet fraction, we selected proteins present only in the insoluble fraction, to discriminate the incidental contamination of soluble proteins, such as the abundant axonemal alpha- and beta-tubulin, which are also present around 55 kDa (Fig 1A) (S7A Fig). Fifteen proteins were identified exclusively from the SDS-resistant pellet of sperm extract (Fig 1B). Among them CG9314, CG4095, Bb8 and Skap proteins show testis-specific expressions based on FlyAtlas and RNA-Seq results [13,15,17]. In addition, CG9314, CG4095 and Bb8 were identified previously in the proteome analysis of *Drosophila melanogaster* sperm [10]. It was recently shown that the testis-specific glutamate-dehydrogenase, Bb8, is required for defining the identity of the mitochondrial derivatives and also part of the testis proteome [12,18]. Skap or A-Sβ function is also described in spermatogenesis, as a mitochondrial enzyme required for caspase activation during spermatid individualization [19]. So far, no function for CG9314 and CG4095 has been described in spermatogenesis; however, both proteins are also present in the sperm proteome. Remarkably, the most abundant proteins in the SDS-resistant 55 kDa fraction were all eight members of the S-Lap protein family (Fig 1B). All S-Laps have a molecular mass around 50–60 kDa and were represented in the mass spectrometry dataset with high peptide counts. Although S-Lap proteins were identified in three previous proteomic analyses of Drosophila sperm, and it is known that their expression is testis-specific, their molecular role in spermatogenesis is unknown [10, 11, 12]. The amount and the chemical features of S-Lap proteins imply that this protein family could contribute to the formation of paracrystalline material in *Drosophila melanogaster*.

### Genetic and functional analysis of S-Lap mutants

**Fig 2:**
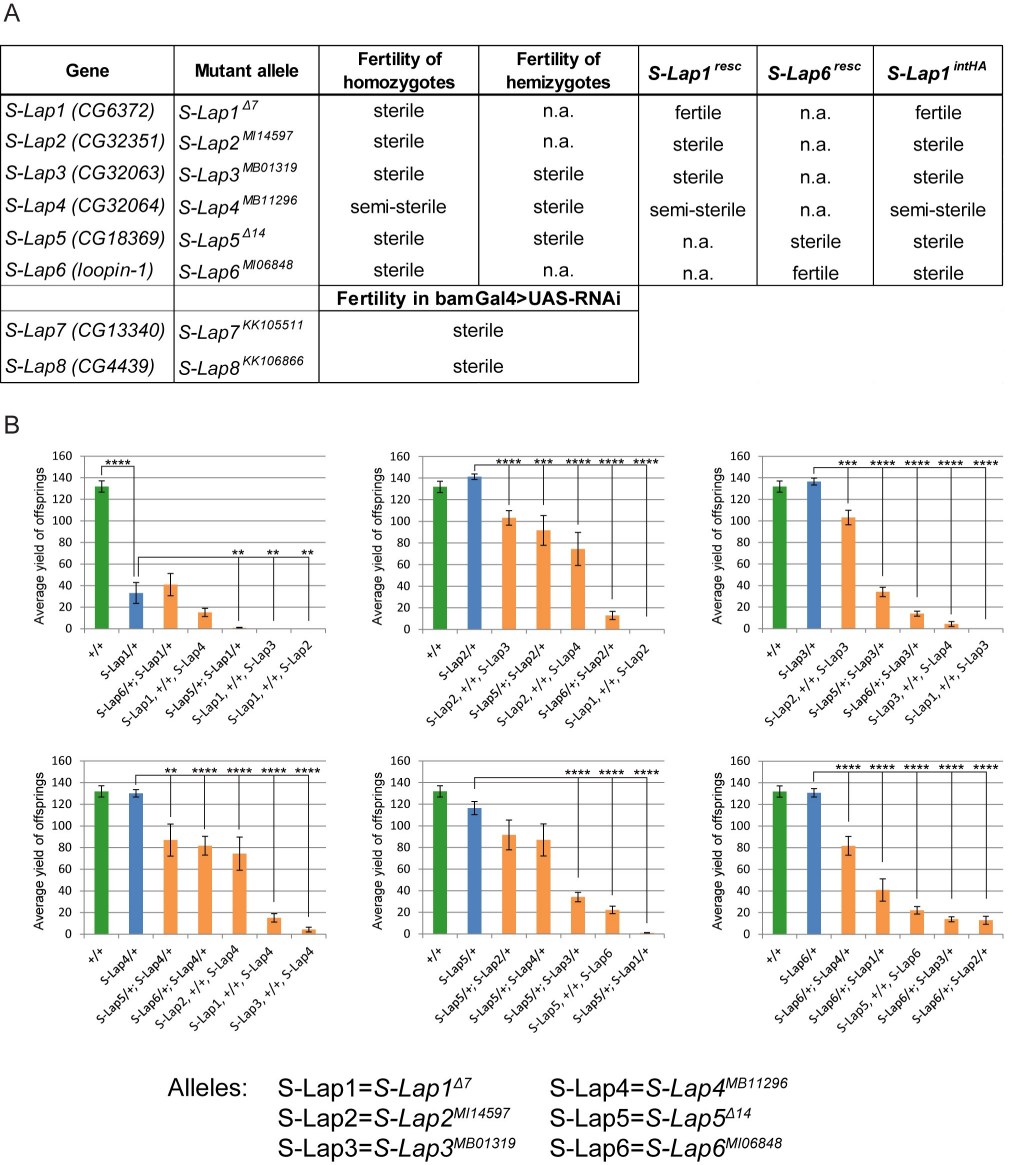
Phenotypes of the mutant alleles of S-Lap genes. (A) Phenotypes of classical mutants (*S-Lap1*^*Δ7*^, *S-Lap2*^*MI14597*^, *S-Lap3*^*MB01319*^, *S-Lap4*^*MB11296*^, S-Lap5^*Δ14*^, *S-Lap6*^*MI06848*^) and RNAi lines (*S-Lap7*^*KK105511*^, *S-Lap8*^*KK106866*^) driven by *bam*-Gal4 testis-specific driver. (B) Diagrams show the average progeny yield of the S-Lap alleles in wild type (green), heterozygotes (blue) and in the transheterozygous (orange) combination. *S-Lap1*^*Δ7*^ allele shows a stronger reduction in progeny number among heterozygotes and gives no more than 40 offspring in average in genetic combinations with other S-Lap alleles. Error bars indicate mean +s.e.m., n = 15 **p<0.01, ***p<0.001, ****p<0.0001 (Student’s t-test).

To test the mutant phenotypes caused by the lack of S-Lap proteins, we collected the publicly available mutants for *S-Lap2*, *S-Lap3*, *S-Lap4* and *S-Lap6* genes and found that in all cases homozygotes were male sterile or semi-sterile (Fig 2A).

For *S-Lap1* and *S-Lap5* genes, we have generated null mutant alleles using the CRISPR-Cas9 method by inducing small deletions, which lead to frameshift mutations in the coding region of the genes. Homozygous males of *S-Lap1*^*Δ7*^ or *S-Lap5*^*Δ14*^ alleles are both male sterile (Fig 2A). Moreover, we observed reduced male fertility in the heterozygotes of the *S-Lap1*^*Δ7*^ allele, suggesting that the *S-Lap1* gene is haploinsufficient (Fig 2B). In the case of *S-Lap3*^*MB01319*^, *S-Lap4*^MB11296^ and *S-Lap5*^*Δ14*^ mutant alleles we analyzed the phenotypes of mutation in trans to deficiency (*S-Lap3*^*MB01319*^/*Df(3L)ED4457*, *S-Lap4*^MB11296^/*Df(3L)ED4457 and S-Lap5*^*Δ14*^/*Df(2R)CX1)* and similarly to the homozygotes we observed the male sterile phenotype (Fig 2A). In the case of *S-Lap7* and *S-Lap8* genes, where classical mutants were not available, we used RNAi-mediated knockdown to reduce gene activity in the male germline. Both *S-Lap7* and *S-Lap8* RNAi resulted in male sterility in the progeny containing the RNAi construct and the germline-specific *bam*-Gal4 driver (Fig 2A). Our results revealed that the lack of a single S-Lap protein is sufficient to cause male sterility, indicating that each of them is crucially important and there is no redundancy in their function.

To analyze the cooperation between S-Lap proteins we also tested the fertility of the transheterozygous combination of the mutant alleles of *S-Lap* genes. We found enhancement of sterile phenotype of *S-Lap1*^*Δ7*^ in the case of heterozygous *S-Lap1*^*Δ7*^, *+*/+, *S-Lap2*^*MI14597*^, *S-Lap1*^*Δ7*^, *+*/+, *S-Lap3*^*MB01319*^, *S-Lap1*^*Δ7*^, *+*/+, *S-Lap4*^*MB11296*^, *S-Lap5*^*Δ14*^/+; *S-Lap1*^*Δ7*^/+ combinations compared to the appropriate heterozygotes (Fig 2B). In addition to the interaction with *S-Lap1*^*Δ7*^ allele, *S-Lap2*^*MI14597*^ also shows a dominant genetic interaction with *S-Lap4*^*MB11296*^ and *S-Lap6*^*MI06848*^. We also found genetic interactions between *S-Lap3* and *S-Lap5*, *S-Lap6* and *S-Lap4*, *S-Lap5* and *S-Lap6* genes in transheterozygotes. The genetic interaction between the S-Lap genes implies that they cooperate in the same process during spermatogenesis.

Previous phylogenetic analysis of S-Lap protein sequences revealed that residues responsible for the leucyl aminopeptidase activity diverged during evolution, and suggested that S-Lap proteins are enzymatically inactive [16]. The classical mutants enabled us to measure the leucyl aminopeptidase activity in testis samples of wild type and different *S-Lap* homozygous alleles, using a specific fluorogenic substrate of leucyl aminopeptidases. We found no reduction in leucyl aminopeptidase activity in any of the mutant testis samples compared to the wild type (S1A Fig). These results demonstrate that lack of a single S-Lap protein does not significantly affect the leucyl aminopeptidase activity of the testis, and also indicate that the male sterility of the mutants is not the consequence of reduced overall aminopeptidase activity.

To directly test the enzymatic activity of S-Lap proteins we decided to express recombinant *S*-*Lap1* and *S-Lap6* and the putative Drosophila M17 aminopeptidase *granny smith* (*grsm*) in bacteria. S-Lap1 differs in three amino acids, while S-Lap6 in six amino acids from the M17 aminopeptidase consensus sequence at the divalent cation binding site and catalytic residues, therefore representing, respectively, a close and distant member of the phylogenetic tree of the Drosophila Leucyl aminopeptidase family [16]. We used the purified 6xHis-tagged Grsm, S-Lap1, and S-Lap6 proteins in the leucyl aminopeptidase activity measurement, and found activity only with Grsm, while both S-Lap1 and S-Lap6 were enzymatically inactive (S1B and S1C Fig). These results provide further evidence that the S-Lap proteins lost their enzymatic activity during evolution and may have another role in spermatogenesis.

### Phenotypic characterization of S-Lap mutants

**Fig 3:**
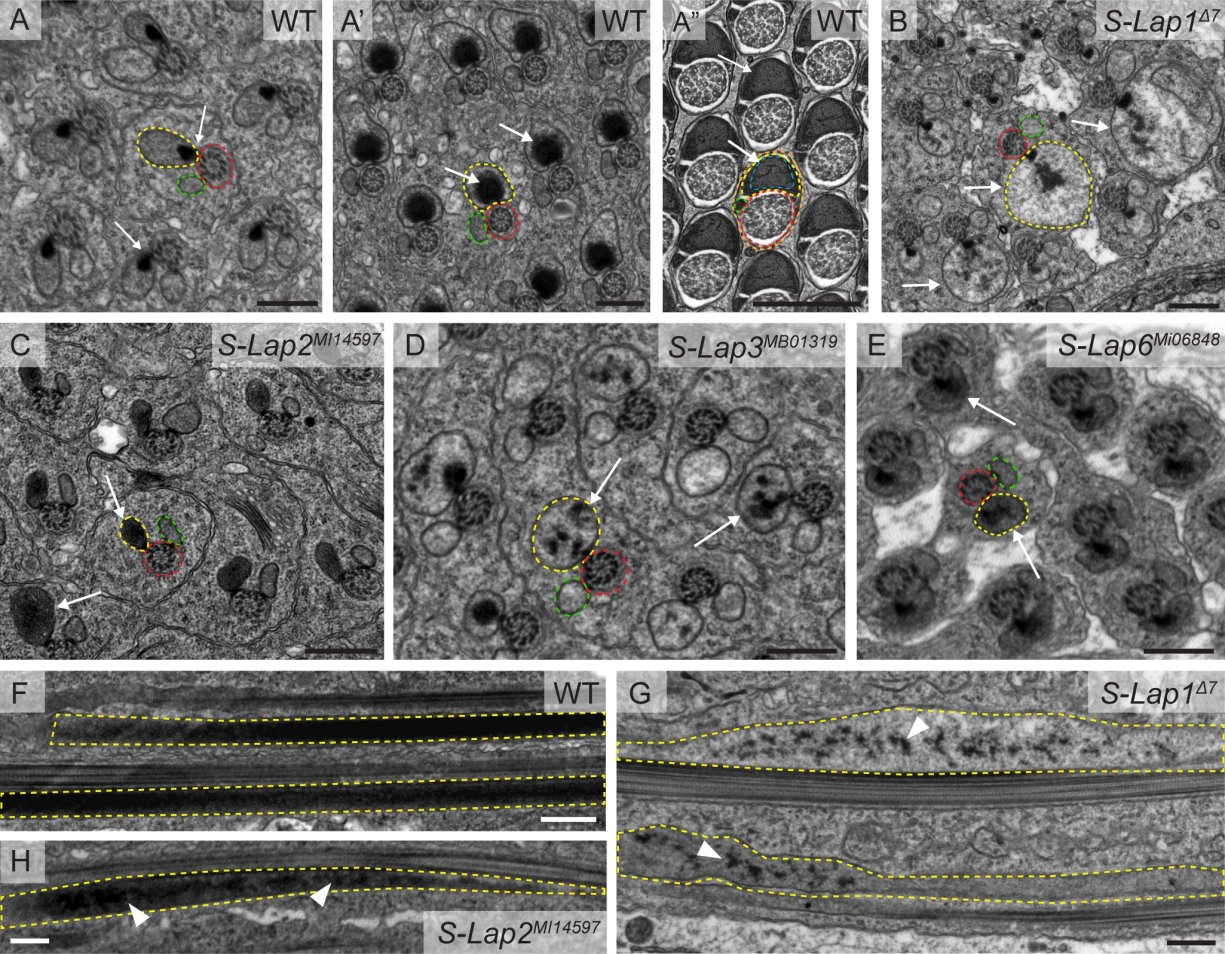
Paracrystalline material formation defects in the S-Lap mutants. (A) Wild-type spermatids show synchronized development of their mitochondrial derivatives in the cysts, where paracrystalline material starts to form at the attachment site of the axoneme (red dashed line) and the major mitochondrial derivative (yellow dashed line) (arrows in A), then accumulate in the growing mitochondria, as a contiguous dense material (arrows in A’), finally it fills the inner space in regularly arranged structure by the end of individualization (arrows in A”), while the minor mitochondrial derivative (green dashed line) regresses (A”). (B-E) The S-Lap mutants (*S-Lap1*^*Δ7*^, *S-Lap2*^*MI14597*^, *S-Lap6*^*MI06848*^) show unsynchronized mitochondrial development with unstructured electron dense paracrystalline material in the major mitochondrial derivatives of spermatids (arrows in B, C, D, E). Scale bars: 0.5 µm. (E-G) Transmission electron micrographs of the longitudinal sections of elongated spermatids in wild type (F), *S-Lap1*^*Δ7*^ (G) and *S-Lap2*^*MI14597*^ (H) mutant testes. The paracrystalline material is solid and unified inside the major mitochondrial derivative (yellow dashed line) in wild type (F), but scattered in the S-Lap mutants (G, H, arrowheads). Scale bars: 0.5 μm.

To determine the precise role of S-Laps in sperm formation, we systematically tested the stages of spermatogenesis in the *S-Lap* mutants and observed no abnormality of spermatogenesis until the onset of meiotic stages. The cell divisions, formation and number of nebenkern were similar to the wild type in all *S-Lap* mutants (S2 Fig). Elongation of the cysts were tested by measuring the length of the elongated cysts and found normal in each mutant (S2, S3A and S3B Figs). In order to get information about late spermatogenesis defects, we tested the establishment of the individualization complexes and the elongation of mitochondria by visualizing them with phalloidin, cleaved-Caspase3 and Mitotracker staining, respectively. We found normal establishment of individualization complexes (IC) around the needle shaped nuclei in all *S-Lap* mutants, but *S-Lap5*^*Δ14*^ mutants contain less IC than the wild type (S4A and S4A’ Fig). We counted the migrating IC and tested the movement of them. We observed that the migration starts in all *S-Lap* mutants, but the number of the migrating IC is generally less than in wild type in all *S-Lap* mutants, with the exception of *S-Lap2*^*MI14597*^ (S4A and S4A” Fig). We found occasionally asynchronized movement of the IC in *S-Lap1*^*Δ7*^, *S-Lap4*^*MB11296*^, S*-Lap6*^*MI06848*^ and it was more severe in *S-Lap2*^*MI14597*^, *S-Lap3*^*MB01319*^, *S-Lap5*^*Δ14*^ and bamGal4>*S-Lap7*^*KK105511*^, bamGal4>*S-Lap8*^*KK106866*^ mutants (S4A, S4A”’ and S4B Fig). In the semi-sterile *S-Lap4*^*MB11296*^ mutants, we observed very few mature sperm in the seminal vesicle. Caspase activation during Drosophila spermatogenesis is connected with individualization and mitochondrial organization [19, 20, 21]. We investigated caspase activation with immunostaining and found a normal cleaved-Caspase-3 signal at the onset of spermatid individualization in all S-Lap mutants (S5A Fig). The elongated mitochondria of *S-Lap1*^*Δ7*^, *S-Lap4*^*MB11296*^, *S-Lap5*^*Δ14*^, S*-Lap6*^*MI06848*^, bamGal4>*S-Lap7*^*KK105511*^ and bamGal4>*S-Lap8*^*KK106866*^ mutants were positive with membrane potential sensitive Mitotracker staining; however, in *S-Lap2*^*MI14597*^ and *S-Lap3*^*MB01319*^ mutants also showed a slightly dashed pattern with Mitotracker (S4B Fig). These data suggests that cyst elongation and overall activity of the mitochondria are not substantially disturbed in the S-Lap mutants. To visualize the post-meiotic elongated mitochondria of the developing spermatids with fluorescent microscopy, we tested the localization of the testis-specific Don Juan-GFP (DJ-GFP), which is localized to the elongated mitochondria of the spermatids [22]. At the onset of spermatid individualization, wild-type spermatids accumulate Don Juan-GFP and this accumulation persists in the late stages of spermatogenesis and in the mature sperm (S5B Fig) [19]. Generally, a weaker or dashed DJ-GFP signal was observed in the *S-Lap1*^*Δ7*^, *S-Lap2*^*MI14597*^ and *S-Lap3*^*MB01319*^ mutants (S5B Fig). Don Juan-GFP was shown to be present in the inner compartments of the major mitochondrial derivative in the elongated spermatids by immuno-EM; therefore the dashed appearance of the Mitotracker and DJ signals in the S-Lap mutants indicates a structural abnormality of the major mitochondrial derivatives [19]. To test this hypothesis, we undertook a more detailed phenotypic characterization of four classical mutants, *S-Lap1*^*Δ7*^ and *S-Lap2*^*MI14597*^, *S-Lap3*^*MB01319*^ and S*-Lap6*^*MI06848*^ by transmission electron microscopy (TEM). This way, we were able to visualize the major structures of the elongated spermatids: axoneme, mitochondria, and microtubules. In wild-type testis, following mitochondrial elongation, only the major mitochondrial derivative of each elongated spermatid accumulates the electron dense paracrystalline material in its lumen and the minor one is reduced in size (Fig 3A, 3A’ and 3A”). In the cross-section of mutant testes of *S-Lap1*^*Δ7*^, *S-Lap2*^*MI14597*^, *S-Lap3*^*MB01319*^ and S*-Lap6*^*MI06848*^ we observed normal axoneme structures with the 9 outer and a central pair of doublet microtubules (Fig 3B, 3C, 3D and 3E). The formation of the two mitochondrial derivatives and the start of paracrystalline material accumulation in the major mitochondrial derivative are completed in the *S-Lap1*^*Δ7*^, *S-Lap2*^*MI14597*^ *S-Lap3*^*MB01319*^ and S*-Lap6*^*MI06848*^ mutants; however, the paracrystalline material is disorganized in all of the tested mutants (Fig 3B, 3C, 3D and 3E). It is worth noting the correlation between the dashed appearance of mitochondria by Mitotracker staining and the degree of paracrystalline abnormality by TEM in the case of *S-Lap1*^*Δ7*^, *S-Lap2*^*MI14597*^ *S-Lap3*^*MB01319*^ mutants (Figs 3B, 3C and 3D and S4B). The sizes of the major mitochondrial derivatives of the examined S-Lap mutants are different from the wild type and the dense paracrystalline material is dispersed inside them (Fig 3B and 3D, arrows). In the longitudinal section of the wild-type testis, we observed paracrystalline material accumulation near the axoneme in the elongated spermatids (Fig 3F). Similarly to the cross-section of the mutants, we observed irregular paracrystalline material in the longitudinal section of the mitochondrial derivatives of *S-Lap1*^*Δ7*^ and *S-Lap2*^*MI14597*^ mutant cysts (Fig 3G and 3H). Overall, TEM analysis demonstrates that the elongated mitochondria are abnormal, with dispersed paracrystalline material in the examined S-Lap mutants. The abnormal mitochondrial morphology could cause abnormal movement of the individualization complexes during individualization in those S-Lap mutants that exhibit dispersed individualization complexes.

### S-Lap proteins localize to the elongated mitochondria

**Fig 4:**
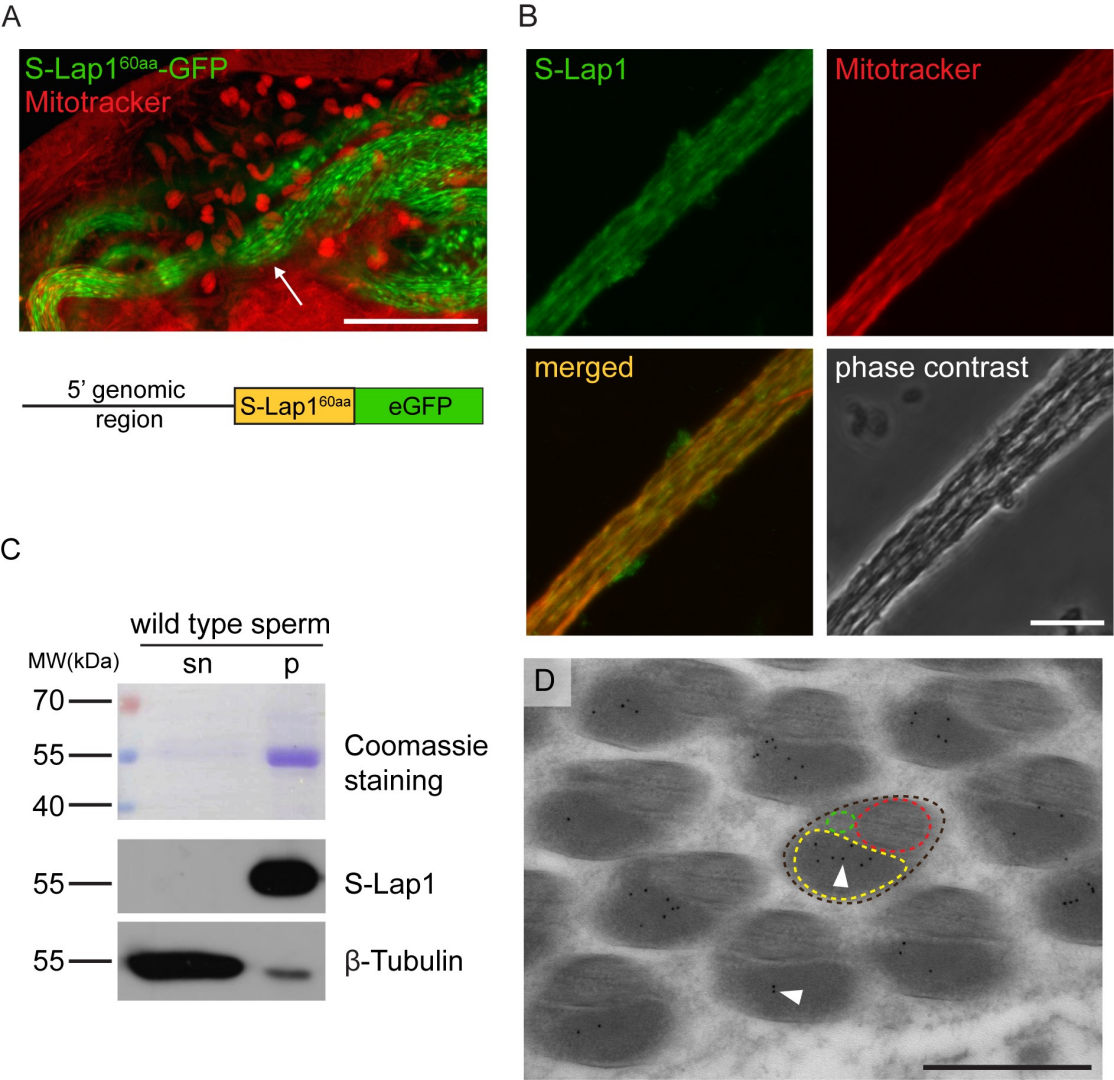
S-Lap1 is localized to the paracrystalline material of the elongated mitochondria. (A) Expression of the genomic reporter construct S-Lap1^60aa-GFP^ (green) shows localization in elongated mitochondria (arrow) by staining with Mitotracker (red). Scale bar: 50 μm. The bottom schematic diagram shows the structure of the S-Lap1^60aa-GFP^ construct with the 5’ genomic region, the mitochondrial targeting sequence coding 60 amino acids and the eGFP sequence. (B) Localization of S-Lap1 protein in elongated mitochondria stained with S-Lap1 antibody (green) and Mitotracker (red) in the squash preparation of wild-type testis. Scale bar: 10 μm. (C) Coomassie Brilliant Blue stained gel and immunoblots of the SDS-soluble supernatant (sn) and the SDS-resistant pellet (p) fraction of wild-type sperm lysates. S-Lap1 is present in the insoluble pellet fraction and β-Tubulin mainly in the soluble fraction of the extract. (D) Representative immuno-electron micrograph of a cross-section of wild-type elongated spermatids (brown dashed line) shows the presence of S-Lap1 protein inside the major mitochondrial derivative (yellow dashed line), represented by gold particles (black dots, arrowheads). Axoneme (red dashed line) and the minor mitochondrial derivative (green dashed line) are also shown. Scale bar: 0.5 μm.

In order to distinguish the direct or indirect effects of S-Lap proteins in mitochondrial development, we tested S-Lap subcellular localization with different approaches. First, S-Lap proteins were analyzed *in silico* by MitoFates software to predict mitochondrial localization signals [23]. N-terminal mitochondrial targeting sequences (MTS) were identified with a mitochondrial processing peptidase (MPP) cleavage site in all eight S-Lap proteins (S7B Fig). In order to test the mitochondrial localization of S-Lap proteins, we fused the first 60 N-terminal amino acid with the predicted mitochondrial targeting sequence of S-Lap1 (S-Lap1^60aa^) protein with eGFP. The expression of the fusion protein was driven by *S-Lap1* upstream genomic region. We observed post-meiotic mitochondrial localization of S-Lap1^60aa^-GFP in the elongated cysts, but it is not present in the mitochondria before meiosis (Fig 4A). These results confirmed the *in silico* prediction of mitochondrial localization of S-Lap1 with an N-terminal mitochondrial targeting sequence and it suggests that other S-Lap proteins could also localize to the elongated mitochondria. This is not surprising in light of the fact that all eight S-Lap transcripts were present in the meiotic and post-meiotic stages of spermatogenesis in a testis region-specific expression analysis of Drosophila testes [14, 16].

Next, we decided to test the detailed subcellular localization of the S-Lap1 protein in developing spermatids with immunostaining using an S-Lap1 antibody. To do this, we compared the primary sequences of all eight S-Lap proteins and found strong amino acid sequence conservation along the entire length of the proteins. The overall identity between the proteins is 53.21%, and each of them has at least one other with higher than 65% identity (S7C Fig). This high level of conservation made the design of a unique epitope region in S-Lap proteins difficult. Thus, we generated a mouse antibody against the full-length S-Lap1 protein knowing that the polyclonal antibody could recognize all the other S-Lap family members too. The pre-immune serum of the S-Lap1 antibody was negative in immunostaining (S6A Fig). We tested the S-Lap1 antibody by immunofluorescence and by Western blot analysis using samples from wild-type, homozygous *S-Lap1*^*Δ7*^ testis and the His-S-Lap1 recombinant protein. We found that the S-Lap1 antibody weakly labels the nebenkern (S5C Fig) and clearly labels the elongated mitochondria of wild-type testis and the signal is strongly reduced in *S-Lap1*^*Δ7*^ mutant (Figs 4B and S5C). S-Lap1 antibody recognizes His-tagged S-Lap1 and S-Lap1 protein from wild-type and *S-Lap1*^*Δ7*^ testis samples and we found that S-Lap1 protein was substantially reduced in the *S-Lap1*^*Δ7*^ testis samples (S5D Fig). The detected protein signal is probably due to the cross-reactivity of the S-Lap1 antibody with the other S-Lap proteins.

### S-Lap1 protein accumulates in the paracrystalline material of the major mitochondrial derivative

We sought to determine the S-Lap1 localization at higher resolution. First, we tested the S-Lap1 antibody by immunoblotting of wild-type sperm samples, where we separated the SDS-soluble supernatant and the SDS-resistant insoluble fraction of protein extract of wild-type sperm. As expected, the S-Lap1 antibody recognizes the processed S-Lap proteins in the predicted ~55 kDa molecular weight range in the pellet fraction, but not in the SDS-soluble supernatant fraction (Fig 4C). Since the S-Lap1 antibody is able to recognize the putative paracrystalline material in the SDS-resistant fraction of sperm protein samples, we wanted to confirm this finding with a different approach. For this, we prepared cross-sections for electron microscopy of wild-type elongated spermatids to test immunocytological localization using the S-Lap1 antibody. We detected S-Lap-specific labeling of gold particles inside the major mitochondrial derivative, where the paracrystalline material is accumulating (Fig 4D). We did not detect labeling with the preimmune serum (S6E Fig). This finding strongly supports the hypothesis that S-Lap proteins could be components of the paracrystalline material.

**Fig 5:**
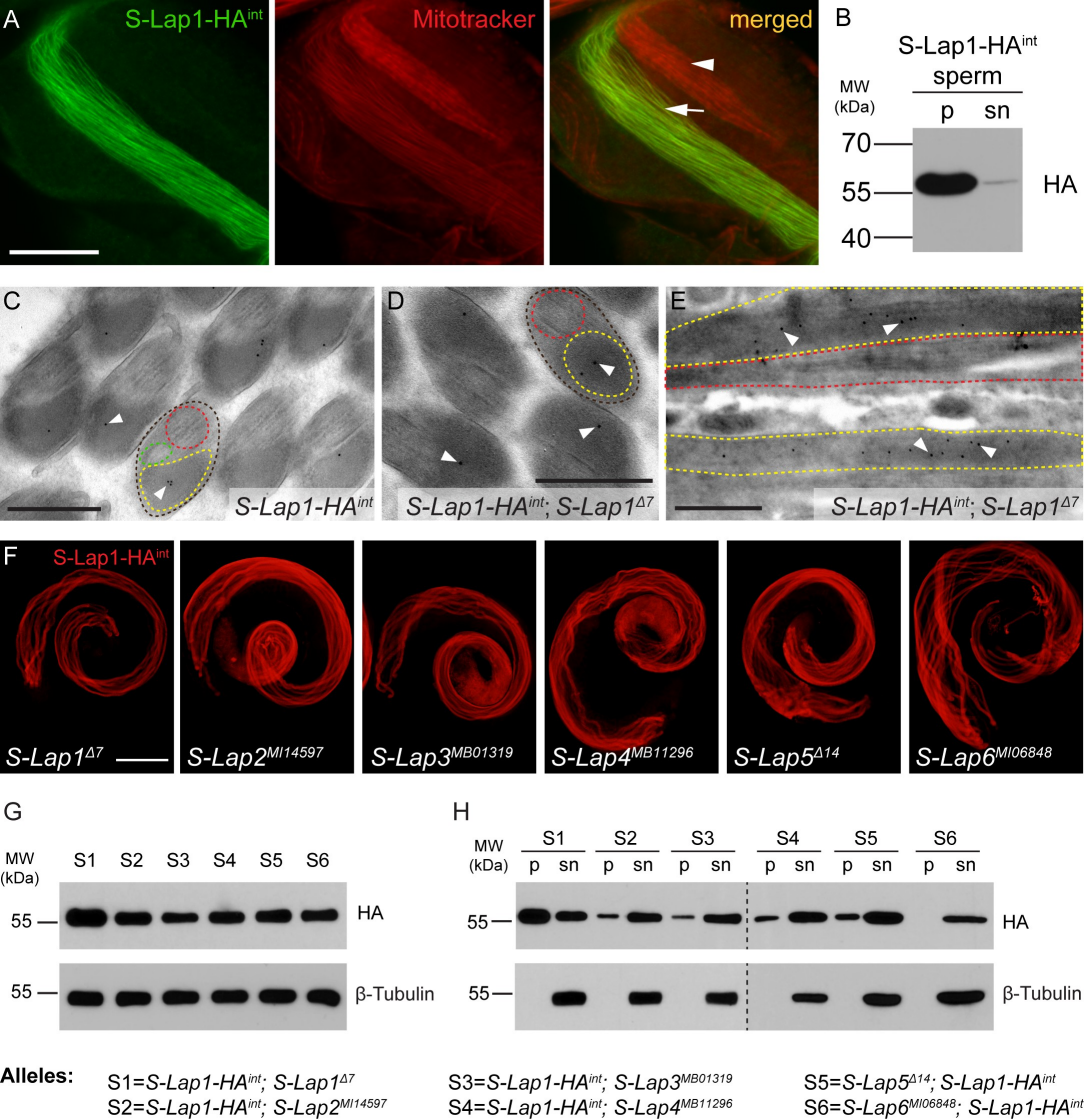
S-Lap1-HA^int^ localizes to the elongated mitochondria. (A) S-Lap1-HA^int^ protein (green) co-localizes (arrow) with the elongated mitochondria of spermatids, but not with the mitochondria of the elongating cyst (arrowhead) (stained with Mitotracker (red)). Scale bar: 10 μm. (B) Immunoblots of the SDS-soluble supernatant (sn) and the SDS-resistant pellet (p) fraction of the transgenic *S-Lap1-HA*^*int*^ sperm lysates using HA antibody. S-Lap1-HA^int^ is mainly present in the insoluble fraction of sperm lysate. (C) Immuno-electron micrograph of a cross-section of transgenic *S-Lap1-HA*^*int*^ elongated spermatids (brown dashed line) shows the occurrence of S-Lap1-HA^int^ protein inside the major mitochondrial derivative (yellow dashed line), represented by gold particles (black dots, arrowheads). Axoneme (red dashed line) and the minor mitochondrial derivative (green dashed line) are also shown. Scale bar: 0.5 μm. (D-E) Cross-section (D) and longitudinal section (E) of immuno-labeled micrographs show S-Lap1-HA^int^ protein accumulation inside the major mitochondrial derivative (yellow dashed line) in spermatids from homozygous *S-Lap1-HA*^*int*^; *S-Lap1*^*Δ7*^ testes, represented by gold particles (black dots, arrowheads). Spermatids (brown dashed line) and axoneme (red dashed line) are labeled. Scale bars: 0.5 μm. (F) S-Lap1-HA^int^ localizes to the elongated mitochondria in testis of *S-Lap1*^*Δ7*^, *S-Lap2*^*MI14597*^, *S-Lap3*^*MB01319*^, *S-Lap4*^*MB11296*^, S-Lap5^*Δ14*^, *S-Lap6*^*MI06848*^ mutants. Scale bar: 200 μm. (G) Immunoblots of total lysate, (H) the SDS-soluble supernatant (sn) and the SDS-resistant pellet (p) fraction of testis expressing the homozygous *S-Lap1-HA*^*int*^ transgene in the classical mutant backgrounds (*S-Lap1*^*Δ7*^, *S-Lap2*^*MI14597*^, *S-Lap3*^*MB01319*^, *S-Lap4*^*MB11296*^, S-Lap5^*Δ14*^, *S-Lap6*^*MI06848*^) using HA antibody. One lane represents 1 testis equivalent protein extract.

To strengthen our findings and to prove the individual participation of S-Lap1 protein in the paracrystalline material, we decided to test the localization of the tagged S-Lap1 protein. First, we generated a genomic rescue construct for *S-Lap1* containing the full genomic region and its promoter region (*S-Lap1*^*resc*^), which enabled S-Lap1 expression in the endogenous pattern. The established transgenic line rescued the male sterility of the *S-Lap1*^*Δ7*^ mutant line (S6B Fig). Next, we modified the *S-Lap1*^*resc*^ vector by tagging S-Lap1 protein with C-terminal hemagglutinin (HA) tag. We tested both the localization and the rescuing capacity of the transgene. Despite the mitochondrial localization, *S-Lap1*^*resc*^*-HA* construct was not able to rescue the male sterile phenotype of the *S-Lap1*^*Δ7*^ mutant, indicating that the C-terminal tag may modify the structure of the protein, which could prevent its normal function. We decided to tag S-Lap1 protein internally with 1xHA on the basis of their predicted tertiary protein structure. We visualized and analyzed the molecular structures of the Drosophila S-Lap proteins using the template of the crystal structure of the bovine lens leucine aminopeptidase (blLAP) [24]. We found that all the eight S-Lap proteins can be aligned to the blLAP model, suggesting a conserved secondary and tertiary protein structure (S6C Fig). Based on the molecular modeling of S-Lap1, we engineered its tagged version, where S-Lap1 protein contains an internal HA insertion right after the amino acid at position 51 (S6D Fig). The expression of the *S-Lap1-HA*^*int*^ transgene fusing its endogenous regulatory region perfectly rescued the male sterile phenotype of homozygous *S-Lap1*^*Δ7*^ mutation (Fig 2A). The S-Lap1-HA^int^ localized to the elongated mitochondria of spermatids as revealed with HA antibody (Fig 5A).

**Fig 6:**
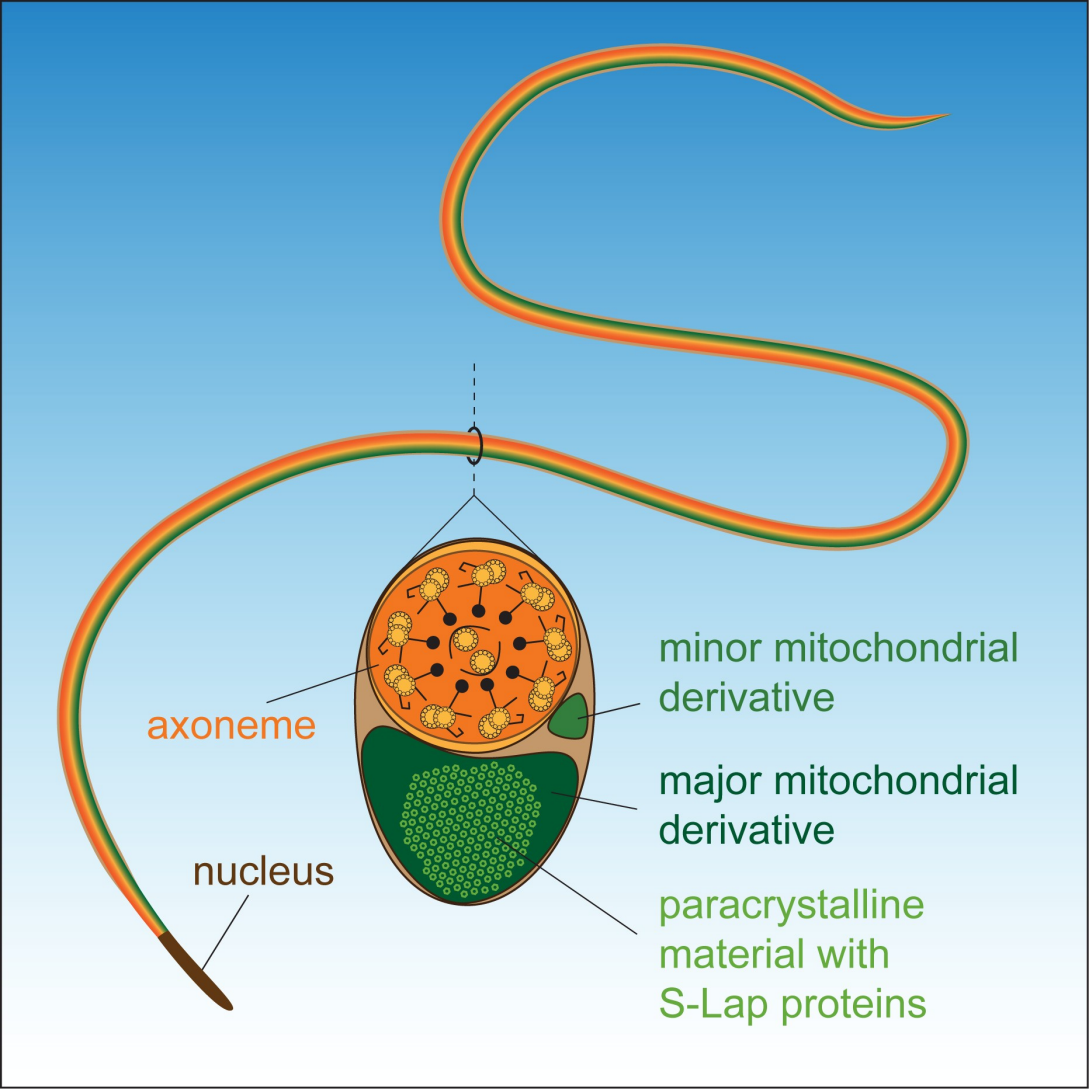
Schematic structure of the *Drosophila melanogaster* sperm.

Moreover, we have also found S-Lap1-HA^int^ tagged protein mostly in the SDS-resistant fraction of isolated sperm from the *S-Lap1-HA*^*int*^ transgenic strain (Fig 5B). Finally, we tested the localization of HA by immunogold staining in cross-section of the adult testis of transgenic flies carrying S-Lap1-HA^int^. We showed the S-Lap1-HA^int^ protein localized to the paracrystalline material of the major mitochondrial derivative of the elongated spermatids in the transgenic line and also in the rescued *S-Lap1-HA*^*int*^; *S-Lap1*^*Δ7*^ mutant line (Fig 5C–5E). We did not get immunogold signal using anti-HA antibody on wild-type testis samples (S6F Fig). To further investigate a possible redundancy between the S-Lap proteins, we overexpressed S-Lap1 (S-Lap1^resc^ and S-Lap1-HA^int^) and also the untagged S-Lap6 (S-Lap6^resc^), in the background of the classical S-Lap mutants (*S-Lap1*^*Δ7*^, *S-Lap2*^*MI14597*^, *S-Lap3*^*MB01319*^, *S-Lap4*^*MB11296*^, *S-Lap5*^*Δ14*^ and S*-Lap6*^*MI06848*^). We found that S-Lap1^resc^, S-Lap1-HA^int^, and S-Lap6 were able to rescue the *S-Lap1*^*Δ7*^ or S*-Lap6*^*MI06848*^ respectively, but not the other S-Lap mutants. This further supports that S-Lap proteins are not redundant and they are not able to complement each other (Fig 2A). We observed the *S-Lap1-HA*^*int*^ transgene exhibited mitochondrial localization in all S-Lap mutant backgrounds, showing that S-Lap1-HA^int^ transport into the mitochondria is not affected by the S-Lap mutations (Fig 5F). S-Lap1-HA^int^ protein is present in a similar amount in all S-Lap mutants (*S-Lap1*^*Δ7*^, *S-Lap2*^*MI14597*^, *S-Lap3*^*MB01319*^, *S-Lap4*^*MB11296*^, *S-Lap5*^*Δ14*,^ and S*-Lap6*^*MI06848*^), when both the individual S-Lap mutations and the *S-Lap1-HA*^*int*^ are homozygous (Fig 5G), however we observed a slight increase in the level of S-Lap1-HA^int^ in *S-Lap1*^*Δ7*^ mutant background. This could be due to the wild type paracrystalline material formation in *S-Lap1-HA*^*int*^; *S-Lap1*^*Δ7*^, which is absent in the other S-Lap mutants in the presence of S-Lap1-HA^int^. To test SDS-resistant paracrystalline material formation, we purified the supernatant and pellet fraction of the SDS treated testis extract of the homozygous S-Lap mutants in the presence of the homozygous *S-Lap1-HA*^*int*^ transgene. We found the S-Lap1-HA^int^ protein both in the supernatant and the pellet fraction in all S-Lap mutants, as the testis sample contains all the differently developed cysts, which represent the different stages of mitochondrial maturation and the organization of paracrystalline material (Fig 5H). S-Lap1-HA^int^ was mainly in the pellet fraction in samples from *S-Lap1-HA*^*int*^/*S-Lap1-HA*^*int*^; *S-Lap1*^*Δ7*^/*S-Lap1*^*Δ7*^, which shows that the tagged protein is able to incorporate into the paracrystalline material (Fig 5H). We found a strong reduction of S-Lap1-HA^int^ in the pellet fraction of testis samples from the S-Lap1-HA^int^-containing *S-Lap2*^*MI14597*^, *S-Lap3*^*MB01319*^, *S-Lap4*^*MB11296*^, *S-Lap5*^*Δ14*,^ and S*-Lap6*^*MI06848*^ mutants, where the S-Lap1-HA^int^ protein is present mainly in the supernatant, suggesting a problem with paracrystalline formation (Fig 5H). These results show that despite the elevation in the amount of S-Lap1 in the other S-Lap mutants, S-Lap1 is not able to complement the other S-Lap proteins, which coincides with the lack of rescue capacity of S-Lap1-HA^int^. Moreover, these results also suggest that the assembly of the proper paracrystalline material relies on the presence of each individual S-Lap protein. Overall, our results indicate that the S-Lap proteins are independently important for spermatogenesis and they are components of the paracrystalline material of the major mitochondrial derivative in *Drosophila melanogaster* sperm (Fig 6).

## Discussion

In this work, we uncovered the S-Lap protein family, whose members are important molecular components of the paracrystalline material of the *Drosophila melanogaster* sperm. The paracrystalline material was first isolated from *Notonecta glauca* and the name crystallomitin was introduced for the constituent proteins. Two components of the paracrystalline material were isolated with molecular weights of 52 and 55 kDa, which show high detergent resistance against SDS and guanidine-HCl extraction. We applied a similar isolation method, in order to identify the components of *Drosophila melanogaster* paracrystalline material by mass spectrometry. The most prominent members of the SDS-resistant pellet fraction of the sperm lysate were all the eight members of the S-Lap protein family. They were previously identified in the Drosophila sperm proteome as the most abundant protein family of the Drosophila sperm [12] with a 2:1 mass ratio when compared to the axonemal constituent tubulin [16]. Based on the fact that mature sperm tail is composed mainly from the axoneme and the mitochondrial derivatives, where the major one is filled with paracrystalline material, and that the S-Lap proteins are the major protein constituents of the sperm proteome, they were good candidates as components of the paracrystalline material [16]. We showed that S-Lap proteins are collectively present in our isolates, S-Laps localize to the major mitochondria, and we found the lack of rescue of the single S-Lap mutants with the overexpression of S-Lap1-HA^int^. These findings all suggest that they could have independent and important structural roles in the formation of the paracrystalline material.

Leucyl aminopeptidases represent a conserved family of the metalloproteases that are able to cleave peptides and proteins at their amino-termini, and they occur in all living organisms, from bacteria to humans, with diverse nomenclature [25]. They have a broad spectrum of functions as cell maintenance enzymes, but many moonlighting functions of LAPs are also elucidated in bacteria and plants [25]. Interestingly, mammalian LAP is present in the bovine and human eye lens, possibly in aggregates, and it may contribute to the formation of lens cataracts by the failure of processing the lens crystallines or by their own precipitation [26, 27]. Despite the importance in pathological conditions, the individual function of leucyl aminopeptidases during spermatogenesis has remained unknown. Proteomic analyses revealed that several members of the LAP proteins are also found to be constituents of sperm across diverse mammalian taxa [28, 29, 30, 31].

The Drosophila Sperm-Leucylaminopeptidases belong to the M17 family with a predicted loss of enzyme activity [16]. Our results show that S-Lap proteins do not contribute individually to the testis aminopeptidase activity; however, they are required individually for normal spermatogenesis, suggesting neofunctionalization of the S-Lap proteins. It is worth mentioning that *granny smith*, the closest paralog of S-Laps, is also expressed in the testis where it could contribute to the leucine cleavage of proteins during spermatogenesis, but its precise role needs to be clarified [32].

We found that the lack of a single S-Lap protein is sufficient to perturb spermatogenesis and overexpression of S-Lap1 or S-Lap6 is not sufficient to rescue the sterility of the other mutants, demonstrating that they do not have redundant functions. We found late spermatogenesis defects in all S-Lap mutants and severe mitochondrial phenotype and abnormal paracrystalline material accumulation in the major mitochondrial derivative in *S-Lap1*^*Δ7*^, *S-Lap2*^*MI14597*^, *S-Lap3*^*MB01319*^ and S*-Lap6*^*MI06848*^ mutants, supporting the involvement of S-Lap proteins in the formation of structured paracrystalline material. Furthermore, we were able to localize S-Lap1 protein to the mitochondria and even more closely to the paracrystalline material of the major mitochondrial derivative of the elongated spermatids.

The first M17 leucine aminopeptidase crystal structure was described in bovine lens and hog kidney [33, 34] and the first model emerged from the bovine eye lens [35]. Since then, the crystal structure of many leucine aminopeptidases, from bacteria to human, have become available in the Protein Data Bank. Based on these studies, all of the M17 LAP proteins form homohexamers. We used homology modeling to build models for all eight S-Lap proteins that show basic structural similarities with the bovine eye lens LAP secondary and tertiary structure. Because of this, we assume that Drosophila S-Lap proteins may be able to build hexamer quaternary structure as well, however, the hexamer formation and the binding of the hexamers with each other or with other proteins need to be determined by further challenging biochemical experiments.

Paracrystalline material can be formed in pathological conditions as well, such as in human mitochondrial myopathies, where creatine kinase (CK) containing paracrystalline material inclusions accumulates in the mitochondria of human skeletal muscle. It can be the result of a compensatory upregulation of CK due to a deficiency of creatine phosphate shuttle activity [36].

It is also worth mentioning the phenomenon that known cytoskeletal proteins and also several globular enzymes are able to go through a filamentation process in certain physiological circumstances. The function of the filament formation in these cases is suspected to be a regulatory mechanism for enzyme activity. One of the most studied examples is cytoplasmic CTP synthase (CTPS), which converts UTP to CTP in the pyrimidine synthesis pathway [37]. CTPS can compartmentalize into filamentous cytoplasmic structures, also termed cytoophydia, in bacteria, yeast and Drosophila cells as well [38, 39, 40]. Filament formation of CTPS is a mechanism to regulate enzyme activity, but novel structural functions of cytoophidia are also speculated, such as a scaffold for biochemical activities or traffic regulation between cellular compartments (reviewed in [37]). More and more enzymes capable of forming filaments are being identified [37, 41]. Interestingly, one of them is the mammalian glutamate dehydrogenase GDH, which is able to polymerize into a quaternary crystalline structure in a concentration-dependent manner in bovine liver cells [42] or form filaments in *Saccharomyces cerevisiae* [41]. We found the testis-specific Drosophila homolog of the mitochondrial glutamate dehydrogenase Bb8 protein in the 55 kDa region of the insoluble detergent-resistant fraction (S5A Fig). Its function was described earlier in the establishment of the identity of the two mitochondrial derivatives and the regulation of paracrystalline material accumulation in the major mitochondrial derivative [18]. Identification of Bb8 in the SDS-resistant fraction of the sperm proteome indicates a more direct structural role in paracrystalline material formation than previously suggested and also that the paracrystalline material could contain other proteins than S-Laps. Our results could contribute to the better understanding of the formation of special ordered protein structures, like paracrystalline material, and filamentous enzymes. Further studies could reveal the hierarchy and the mechanism of how the components of the sperm paracrystalline material bind or interact with each other. Summarizing the genetic, molecular and biochemical data, we propose a model in which S-Lap proteins are important structural constituents of the paracrystalline material of the mature sperm of *Drosophila melanogaster* (Fig 6).

## Materials and methods

### Fly stocks, mutants, and fertility test

Flies were maintained on standard cornmeal agar medium at 25°C. Fly stocks used in this study were obtained from Bloomington Drosophila Stock Center: *y*^*1*^ w^*^; Mi{MIC}S-Lap2^*MI14597*^, *y*^*1*^ *w*^*67c23*^*; Mi{ET1}S-Lap3*^*MB01319*^, *w*^*1118*^ *; Mi{ET1}S-Lap4*^*MB11296*^, *y*^*1*^ w^*^; Mi{MIC}loopin-1^*MI06848*^, *w*^*1118*^*; Df(3L)ED4457/TM6C cu*^*1*^ *Sb*^*1*^, *Df(2R)CX1*, *wg*^*12*^ *b*^*1*^ *pr*^*1*^*/SM1*, *w*^*1118*^*; Df(2R)BSC550/CyO* and *SLap7*^*KK105511*^, *S-Lap8*^*KK106866*^ and RNA interference lines from Vienna Drosophila Stock Center [43]. The RNAi lines were crossed with *bam*-Gal4-VP16 driver [44] (kindly provided by Helen White-Cooper).

### Transgenic constructs

The *S-Lap1*^*Δ7*^ allele contains a 7 bp deletion and *S-Lap5*^*Δ14*^ allele contains 14 bp deletion, generated by using the CRISPR-Cas9 method, as described in Port et al. [45]. Briefly, one sgRNA/gene was cloned into the pCFD4 plasmid (Addgene #49411) at Bbs1 site and then injected into a fly line containing a source of φ-31 integrase and an attP landing site, *y1 v1 P{nos-phiC31\int*.*NLS}X; P{CaryP}attP40* (Bloomington Stock Center No: 25709). The sgRNA lines were crossed with the Cas9 source line (*y1 M{nos-Cas9*.P}ZH-2A w^*^) to generate deletions in the target genes. The deletions were screened by a T7 endonuclease I assay, and then sequenced and stable lines were established [46]. The sequences used for generating sgRNA lines are listed in S8A Fig. The *S-Lap1*^*Δ7*^, *S-Lap2*^*MI14597*^, *S-Lap3*^*MB01319*^ alleles were combined with DJ-GFP line w^*^; P{dj-GFP.*S}AS1/CyO* [22].

To make a genomic rescue construct for *S-Lap1* (CG6372) and *S-Lap6* genes, the upstream regions (1332 bp before *S-Lap1* and 2141 bp before *S-Lap6*) and the full gene sequence were amplified with Phusion High-Fidelity DNA Polymerase (Thermo Fisher Scientific) and the PCR fragment was first cloned into a pJET1.2 vector (Thermo Fisher Scientific) and then into a pUASTattB [47] vector using restriction sites NotI and XbaI. To make a tagged version of the S-Lap1 rescue construct, an internal 1xHA insertion was designed (S8B Fig). In site-directed mutagenesis, the whole pJET1.2-S-Lap1 genomic rescue construct was amplified with overlapping primer pairs containing the HA sequence, using Phusion High-Fidelity DNA Polymerase (Thermo Fisher Scientific), then digested with DpnI restriction enzymes to eliminate the original template. The S-Lap-HA^int^ fragment was cloned into a pUASTattB vector using NotI and XbaI sites. The S-Lap1^60aa^-GFP construct was cloned by amplifying the 1332 bp upstream sequence and the first 180 bp from the *S-Lap1* coding sequence, and the fragment was cloned into a modified pUASTattB-GFP vector using NotI-XhoI sites. Primer sequences used for cloning are listed in S8B Fig.

Transgenic constructs were injected into *y1*, *v1*, *P{nos-phiC31\int*.*NLS}X; P{CaryP}attP40*a and *y1*, *sc1*, *v1*, *P{y[+t7*.*7] = nos-phiC31\int*.*NLS}X; P{y[+t7*.*7] = CaryP}attP2* fly lines.

For fertility tests, individual males (minimum 30/genotype) were crossed with three *w*^*1118*^ virgin females. Fourteen days after crossing, hatched progeny was counted in every tube.

### Purification of the detergent-insoluble fraction, mass spectrometry, and immunoblotting

For proteomic analysis and immunoblotting 50 seminal vesicles or 20 pairs of testis of 10 days old flies were dissected and homogenized in 100 μl modified RIPA buffer (140 mM NaCl, 10 mM Tris-HCl pH 8.0, 1 mM EDTA, 0.5 mM EGTA, 1% Triton-X-100, 0.1% sodium deoxycholate, 1% sodium dodecyl sulphate (SDS), 1 mM PMSF). Cellular debris was pelleted at 2000 rpm for 2 minutes, and then the supernatant was centrifuged at 15000 rpm for 15 minutes at 4°C, removed and mixed with 20 μl 6xLaemmli sample buffer. The insoluble pellet was washed two times with 0.75 M Tris-HCl (pH 7.6) and centrifuged at 15000 rcf for 15 minutes, then suspended in 120 μl 1xLaemmli buffer. Samples were separated on 10% SDS polyacrylamide gels (Bio-Rad). For proteomic analysis, the gel was stained with Coomassie Brilliant Blue then destained with the mix of 40% ethanol and 10% acetic acid. The 50-65 kDa region of the soluble and insoluble sample were subjected to in-gel digestion as described in Migh et al. [48]. Briefly, protein disulfide bridges were reduced with dithiothreitol (30 min, 56°C) then the resulting sulfhydryl groups were capped using iodoacetamide (30 min, RT, in the dark) following digestion with sequencing grade side chain protected trypsin (4h, 37°C). The resulting peptide mixtures were analyzed by LC-MS/MS using a Waters nanoAcquity UPLC online coupled to an Orbitrap Elite mass spectrometer (Thermo Fisher Scientific) operating in the positive ion mode. Peptides were separated on a Waters BEH300 C18 column (75 μm ID * 250 mm length, 1.7 μm particle size) by a linear gradient of 10–40% B in 30 minutes (solvent A: 0.1% formic acid in water, solvent B: 0.1% formic acid in acetonitrile; flow rate: 400 nl/min). Mass spectrometric analysis was performed in data-dependent mode acquiring MS/MS (CID, normalized collision energy: 35) spectra of the ten most abundant multiply charged precursor ions following each MS survey scans (m/z range: 380–1400). MS spectra were acquired in the Orbitrap (resolution: 60000), MS/MS spectra in the linear ion trap. Raw data were converted into peak lists using the PAVA software [49] and searched with the ProteinProspector search engine (v.5.16.0) using the UniProt database (2015.12.14 download) with *Drosophila melanogaster* species restriction (42462 protein sequences), each entry was concatenated with a randomized sequence. The following search parameters were applied: mass accuracy: 5 ppm for precursor ions and 0.6 Da for fragment ions; enzyme: trypsin with maximum one missed cleavage site; fixed modifications: carbamidomethyl; variable modifications: Met oxidation, pyroGlu formation from N-terminal Gln residues and acetylation of protein N-terminus; instrument: ion trap. The following acceptance criteria were applied: score, 22 and 15; E-value, 0.01 and 0.05 for protein and peptide identifications, respectively.

For immunoblotting, the protein samples were fractionated by SDS-PAGE and transferred to PVDF membrane. Blocking and antibody incubations were carried out in Tris-buffered saline (Sigma-Aldrich) with 0.05% Tween-20 (TBST) containing 4% nonfat dry milk. Primary antibodies were anti-beta-tubulin E7 (1:1000 dilution, Developmental Studies Hybridoma Bank, anti-HA (1:1000 dilution, Roche), S-Lap1 antibody (1:1000 dilution) and anti-His antibody (1:3000 dilution, Invitrogen, MA1-21315). HRP-linked secondary antibodies (Millipore) were used at a dilution of 1:5000. After incubation with the antibodies, blots were washed in TBST and imaged on X-ray film using an ECL detection kit (GE Healthcare).

### Recombinant protein expression and purification and antibody production

Full-length coding DNA sequences of *Drosophila grsm*, *S-Lap1* and *S-Lap6* were inserted into the pDEST17 plasmid in frame with the N-terminal polyhistidine-tag using the Gateway system (Thermo Fisher Scientific). The constructs were transformed into chemically competent *E*. *coli* SixPack strain [50]. Bacteria were grown for 3 days at 16°C in auto induction medium (AIM-LB with trace elements, Conda), harvested by centrifugation (3000*xg*, 4°C, 15 min), lysed by sonication in ice-cold buffer containing 50 mM NaH_2_PO_4_ pH 8.0, 300 mM NaCl, 20 mM imidazole, 0.1% Triton X-100 and 5% 2-mercaptoethanol (Buffer-1) and centrifuged (21.000*xg*, 4°C, 15 min) to pellet debris. The clarified lysate was incubated with Ni-NTA agarose beads (Qiagen) for 2h at 4°C. Beads were washed twice in Buffer-1, twice in Buffer-2 (Buffer-1 supplemented with 200 mM NaCl) and twice in Buffer-1 followed by the elution of the recombinant proteins in buffer containing 50 mM Tris-HCl pH 8.0, 150 mM NaCl and 300 mM imidazole. Eluates were concentrated and dialyzed against a buffer containing 50 mM Tris-HCl pH 7.5, 150 mM NaCl, 1 mM MnCl_2_ and 50 μM ZnCl_2_. Purified proteins were run on SDS-PAGE and tested by immunoblotting before using in *in vitro* enzyme activity assay. For antibody production, a His-tagged S-Lap1 fusion protein construct was generated in a pET28b vector (Novagen). The transgenic construct was transformed into *Escherichia coli* BL21 strain, and expression was induced with 0.1 M IPTG overnight at 18°C. The S-Lap1-His protein was purified with BugBuster Protein Extraction Reagent (Novagen) following the manufacturer's instructions, and then the S-Lap1 protein was purified from a 10% acrylamide gel according to Michalk et al. [51] and used for immunization of mice (BRC HAS, Szeged, Hungary).

### Leucyl aminopeptidase assay

Testes from 10 males/genotype were dissected and sonicated in 400 μl incubation buffer (50 mM HEPES pH 7.4, 1 mM MnCl_2_, 1mM DTT), and then centrifuged at 15000 rcf at 4°C. Equal amounts of bacterially expressed and purified recombinant His-Grsm, His-S-Lap1 and His-S-Lap6 proteins were used in an *in vitro* aminopeptidase assay. The leucyl aminopeptidase activity was measured from 100 μl of the testis supernatant or purified protein, after adding 100 μM L-Leu-AMC (Sigma-Aldrich), by fluorescence emission at 460 nm (340 nm excitation) using FLUOstar OPTIMA Plate Reader.

### Staining and microscopy

Testis preparation and staining were performed as described earlier by White-Cooper [52]. Texas Red-X Phalloidin and Alexa Fluor 488 Phalloidin (Thermo Fisher Scientific) were used at a 1:250 dilution. Mitotracker Red CMXRos, (Thermo Fisher Scientific) was used at a 0.5 μM concentration diluted in PBS and dissected testes were stained for 20 minutes. 4',6-diamidino-2-phenylindole (DAPI) were used at 1 μg/ml concentration, Texas Red-X Phalloidin (Life Technologies) was used at a dilution of 1:250. Rat anti-HA antibody (1:100) (Roche), rabbit anti-cleaved-Caspase3 (1:200) (clone 5A1E, Cell Signalling), mouse anti-pan polyglycylated Tubulin antibody (1:5000) (clone AXO 49, Merck) and mouse S-Lap1 polyclonal antibody were used at dilutions of 1:200. Alexa Fluor 488 and Alexa Fluor 546 conjugated anti-mouse, anti-rat and anti-rabbit antibodies (Invitrogen) were used as secondary antibodies at a dilution of 1:400. Samples were mounted in SlowFade Gold antifade reagent (Life Technologies). Images were taken by Olympus BX51 fluorescent microscope or Olympus Fluoview Fv10i Confocal microscope. Images were processed with ImageJ and GIMP 2.8.2.

Electron microscopic analyses of testes were done as described earlier [53]. Briefly, for both traditional and immune electron microscopy, testes were dissected and fixed overnight at 4°C in 3.2% paraformaldehyde, 1% glutaraldehyde, 1% sucrose, 0.028% CaCl2 in 0.1 N sodium cacodylate (pH = 7.4), thoroughly washed in 0.1 N sodium cacodylate (pH = 7.4), post-fixed in 0.5% osmium tetroxide for 1h, and embedded in Durcupan (Fluka) resin according to the manufacturer’s recommendations.

For traditional EM 70 nm sections were cut, stained in Reynold’s lead citrate and evaluated using a JEM-1011 electron microscope (JEOL) equipped with a Morada camera and iTEM software (Olympus).

For immunogold labeling, 90 nm sections were treated first with 5% H_2_O_2_ at RT for 1 minute and washed 3x5 minutes in distilled water, then treated with 0.1% NaHB_4_ in TBS buffer (pH 5.5) for 10 min, and finally with 50 mM glycin in TBS for 30 minutes. Next, the samples were washed in TBS buffer for 3x5 minutes and blocked for 30 minutes in 10% FBS, 5% dried milk, 1% BSA diluted in TBS. Mouse anti-S-Lap1 (1:30) and rat anti-HA (1:10) (Roche) antibodies were diluted in solutions containing 5% FBS, 2.5% dried milk, 1% BSA in TBS and applied on the sections overnight at 4°C, then washed 3 times with 2% FBS, 1.25% dried milk, 1% BSA in TBS for 5 min. The primary antibodies were detected by incubating the samples with anti-rat IgG (whole molecule)-gold antibody (Sigma-Aldrich) or anti-mouse IgG (whole molecule)-gold antibody (Sigma-Aldrich), where IgG is conjugated with 10 nm gold particles, in a 1:50 dilution at room temperature for 120 minutes. Sections were washed in TBS 3x5 minutes and put in 1% cacodylate buffered glutaraldehyde for 10 minutes to enhance the attachment of gold particles; rinsing in distilled water 3x5 minutes completed the procedure. Sections were evaluated using a JEM-1011 electron microscope (JEOL) equipped with a Morada camera and iTEM software (Olympus).

### Bioinformatical analysis and molecular modeling

Protein sequence alignment was made using Clustal Omega (EMBL-EBI). Analysis of intracellular localization signal was made by Predotar [54] and then the mitochondrial targeting signal was defined by MitoFates [23]. Comparative structural modeling was carried out using UCSF Chimera [55] and Modeller [56]. Welch two sample t-test was used to determine significance of length measurement data and individualization phenotype data.

## Supporting information

S1 Fig Leucyl aminopeptidase activity of the S-Lap mutants. (A) Diagram shows the relative fluorescence intensity measured in the leucyl aminopeptidase assay from the testes of homozygous S-Lap mutants after adding the fluorescent substrate L-Leu-AMC. No significant reduction in aminopeptidase activity is observed in the S-Lap mutants compared to wild-type (WT). Error bars indicate mean +s.e.m., n = 3. (B) Immunoblot of the purified recombinant His-S-Lap1, His-S-Lap6 and His-Grsm proteins using anti-His antibody. (C) An equal amount of His-S-Lap1, His-S-Lap6 and His-Grsm proteins were tested in the leucyl aminopeptidase assay by measuring the relative fluorescence intensity of the fluorescent substrate L-Leu-AMC for 6 hours.
(TIF)

S2 Fig Phenotypic characterization of S-Lap mutants by phase contrast microscopy and Mitotracker staining. Different stages of spermatogenesis (spermatocytes, round spermatids) in a whole testis in wild type and S-Lap mutants (*S-Lap1*^*Δ7*^, *S-Lap2*^*MI14597*^, *S-Lap3*^*MB01319*^, *S-Lap4*^*MB11296*^, S-Lap5^*Δ14*^, *S-Lap6*^*MI06848*^, bamGal4>*S-Lap7*^*KK105511*^, bamGal4>*S-Lap8*^*KK106866*^) were visualized by nuclear (Hoechst (blue)) and, mitochondrial (Mitotracker (red)) staining and phase contrast microscopy. Scale bars: 20 μm. The whole testis with elongated cysts was visualized with phase contrast microscopy. Scale bars: 200 μm.
(TIF)

S3 Fig Post-meiotic cyst elongation is normal in the S-Lap mutants. (A) Confocal micrographs of elongated spermatid cysts of wild-type (WT) and S-Lap mutants (*S-Lap1*^*Δ7*^, *S-Lap2*^*MI14597*^, *S-Lap3*^*MB01319*^, *S-Lap4*^*MB11296*^, S-Lap5^*Δ14*^, *S-Lap6*^*MI06848*^, bamGal4>*S-Lap7*^*KK105511*^, bamGal4>*S-Lap8*^*KK106866*^) stained with Axo49 tubulin antibody (green) and nuclei with DAPI (blue). Scale bar: 200 μm. (B) The boxplot shows the length of the Axo49 positive cysts in WT and S-Lap mutants.
(TIF)

S4 Fig Individualization complex formation and movement in S-Lap mutants. (A) Individualization complexes were counted on phalloidin stained testes samples in S-Lap mutants. n(testis) represents the number of the analyzed testes. (A’) Needle shaped nuclei with individualization complexes, (A”) migrating individualization complexes and (A”‘) disturbed migrating individualization complexes were counted in WT and S-Lap mutants. Statistical significance was determined by Welch two sample t-test. (B) Confocal micrographs of elongated spermatids in wild type (WT)and S-Lap mutants (*S-Lap1*^*Δ7*^, *S-Lap2*^*MI14597*^, *S-Lap3*^*MB01319*^, *S-Lap4*^*MB11296*^, S-Lap5^*Δ14*^, *S-Lap6*^*MI06848*^, bamGal4>*S-Lap7*^*KK105511*^, bamGal4>*S-Lap8*^*KK106866*^) show elongated mitochondria stained with Mitotracker (red) and investment cones marked by phalloidin (green) with synchronized and unsynchronized movement. Scale bar: 25 μm.
(TIF)

S5 Fig Caspase activation, mitochondrial DJ-GFP distribution in S-Lap mutants. Characterization of the S-Lap1 antibody. (A) Cleaved-Caspase3 (green) is present in the elongated cysts in wild type (WT) and S-Lap mutants (*S-Lap1*^*Δ7*^, *S-Lap2*^*MI14597*^, *S-Lap3*^*MB01319*^, *S-Lap4*^*MB11296*^, S-Lap5^*Δ14*^, *S-Lap6*^*MI06848*^, bamGal4>*S-Lap7*^*KK105511*^, bamGal4>*S-Lap8*^*KK106866*^). Individualization complexes were stained with phalloidin (red) and nuclei with DAPI (blue). Scale bar: 200 μm. (B) Investment cones are synchronized and mitochondrial DJ-GFP distribution is smooth, while in the *S-Lap1*^*Δ7*^ mutant investment cones are synchronized and DJ-GFP distribution is slightly dashed. In the *S-Lap2*^*MI14597*^ mutant and the *S-Lap3*^*MB01319*^ mutant, the investment cones are dispersed and DJ-GFP distribution is dashed. Scale bar: 50 μm. (C) S-Lap1 antibody (red) decorates weakly the nebenkern in round spermatids (arrows) and the elongating and the elongated mitochondria (arrowhead) both in WT and S-Lap1 mutants. The signal intensity is reduced in the *S-Lap1*^*Δ7*^ testis by using the same exposure time as in the WT. Nuclei were stained with DAPI (blue). Scale bars: 200 μm and 50 μm. (D) Immunoblot of WT and *S-Lap1*^*Δ7*^ testes using S-Lap1 antibody, where one lane represents 1 testis equivalent total lysate. S-Lap1 antibody recognizes the recombinant His-S-Lap1 protein. Note that the His-tagged unprocessed recombinant protein runs higher (~70 kDa) than the processed and untagged endogenous S-Lap1 (~55 kDa).
(TIF)

S6 Fig S-Lap1 antibody specificity, rescue of the *S-Lap1*^*Δ7*^, structural models of S-Lap proteins and cryo cross-sections of wild type testis. (A, A’, A”) Confocal micrographs of elongated cysts stained with mouse pre-immune serum of S-Lap1 antibody (green), and Mitotracker (red) in squash preparation of wild-type testis. Scale bar: 10 μm. (B) Phase contrast and fluorescent images (B’, B”) of the testis of the *S-Lap1*^*Δ7*^ mutant expressing the S-Lap1 genomic rescue construct (*S-Lap1*^*resc*^), stained with phalloidin (red) and the nuclei with DAPI (blue). The seminal vesicle contains individualized sperms (arrow on B, grey square on B’ and in larger magnification on B”). Scale bars: 200 μm (B, B’) 50 μm (B”). (C) Three-dimensional models of S-Lap proteins compared to the bovine leucine aminopeptidase protein model 2EWB. (D) Three-dimensional model alignment of S-Lap1 (red) and S-Lap1-HA^int^ (blue) and the position of the internal 1xHA tag (green). (E, F) Cryo cross-sections of wild-type spermatids immuno-labeled with (E) pre-immune serum of S-Lap1 antibody and anti-HA antibody (F) do not show specific labeling after developing with nanogold particles. Brown dashed line borders one elongated spermatid with axoneme (red dashed line) and with major mitochondrial derivative (yellow dashed line) inside. Scale bars: 0.2 μm.
(TIF)

S7 Fig Proteins of isolated paracrystalline, mitochondrial targeting signal sequences and the sequence similarity of S-Lap proteins. (A) Analysis of protein accumulation in the 50-60 kDa molecular weight region of SDS-resistant pellet fraction of sperm extract by mass spectrometry. (B) S-Lap proteins contain an N-terminal mitochondrial target sequence with high probability. The MPP (mitochondrial processing peptidase) cleavage sites were predicted using the online software MitoFates. (C) Sequence similarity between S-Lap proteins. Alignment analysis was made by Clustal Omega software.
(TIF)

S8 Fig Sequences and primers. (A) The sequences used for generating sgRNA lines and (B) the primers used for cloning transgenic constructs.
(TIF)
