# Supplementary figures and images for: Sperm-Leucylaminopeptidases are required for male fertility as structural components of mitochondrial paracrystalline material in Drosophila melanogaster sperm
Source: PLoS Genet. 2019 Feb 25;15(2):e1007987. doi: 10.1371/journal.pgen.1007987 (PMC6388916; doi:10.1371/journal.pgen.1007987)

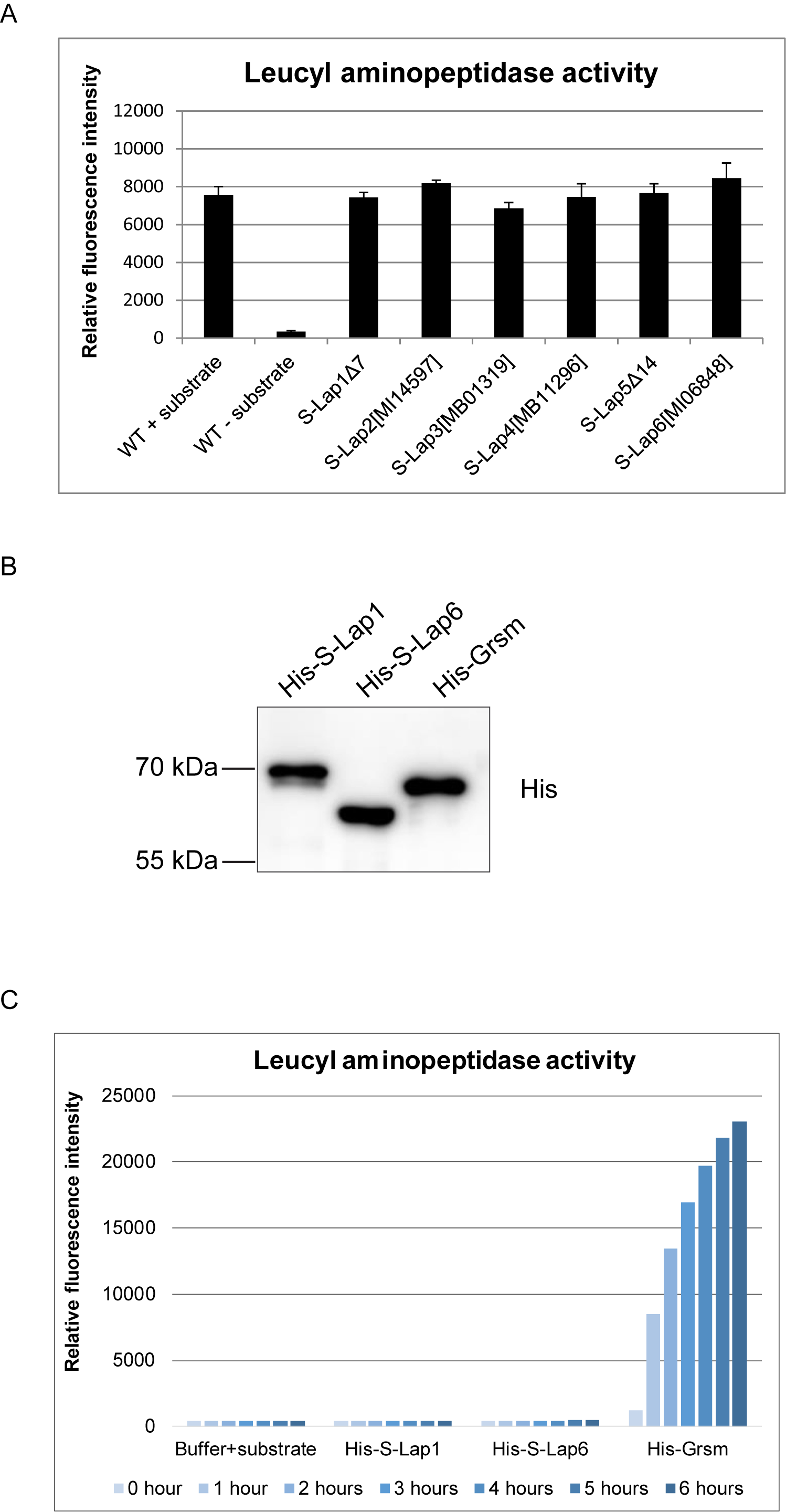

Supplement: S1 Fig — (A) Diagram shows the relative fluorescence intensity measured in the leucyl aminopeptidase assay from the testes of homozygous S-Lap mutants after adding the fluorescent substrate L-Leu-AMC. No significant reduction in aminopeptidase activity is observed in the S-Lap mutants compared to wild-type (WT). Error bars indicate mean +s.e.m., n = 3. (B) Immunoblot of the purified recombinant His-S-Lap1, His-S-Lap6 and His-Grsm proteins using anti-His antibody. (C) An equal amount of His-S-Lap1, His-S-Lap6 and His-Grsm proteins were tested in the leucyl aminopeptidase assay by measuring the relative fluorescence intensity of the fluorescent substrate L-Leu-AMC for 6 hours. (TIF) [file pgen.1007987.s001.tif]

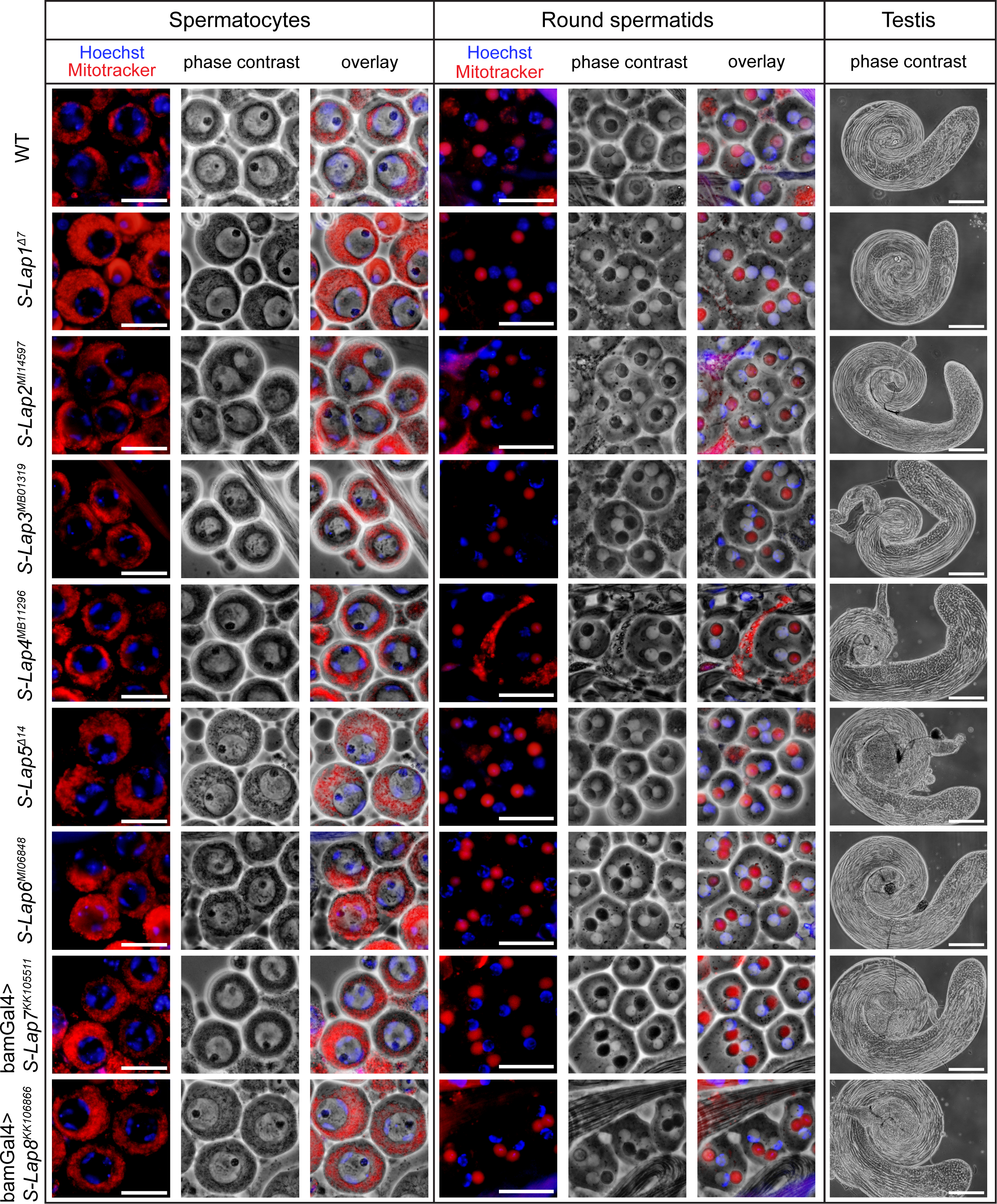

Supplement: S2 Fig — Different stages of spermatogenesis (spermatocytes, round spermatids) in a whole testis in wild type and S-Lap mutants (S-Lap1Δ7, S-Lap2MI14597, S-Lap3MB01319, S-Lap4MB11296, S-Lap5Δ14, S-Lap6MI06848, bamGal4>S-Lap7KK105511, bamGal4>S-Lap8KK106866) were visualized by nuclear (Hoechst (blue)) and, mitochondrial (Mitotracker (red)) staining and phase contrast microscopy. Scale bars: 20 μm. The whole testis with elongated cysts was visualized with phase contrast microscopy. Scale bars: 200 μm. (TIF) [file pgen.1007987.s002.tif]

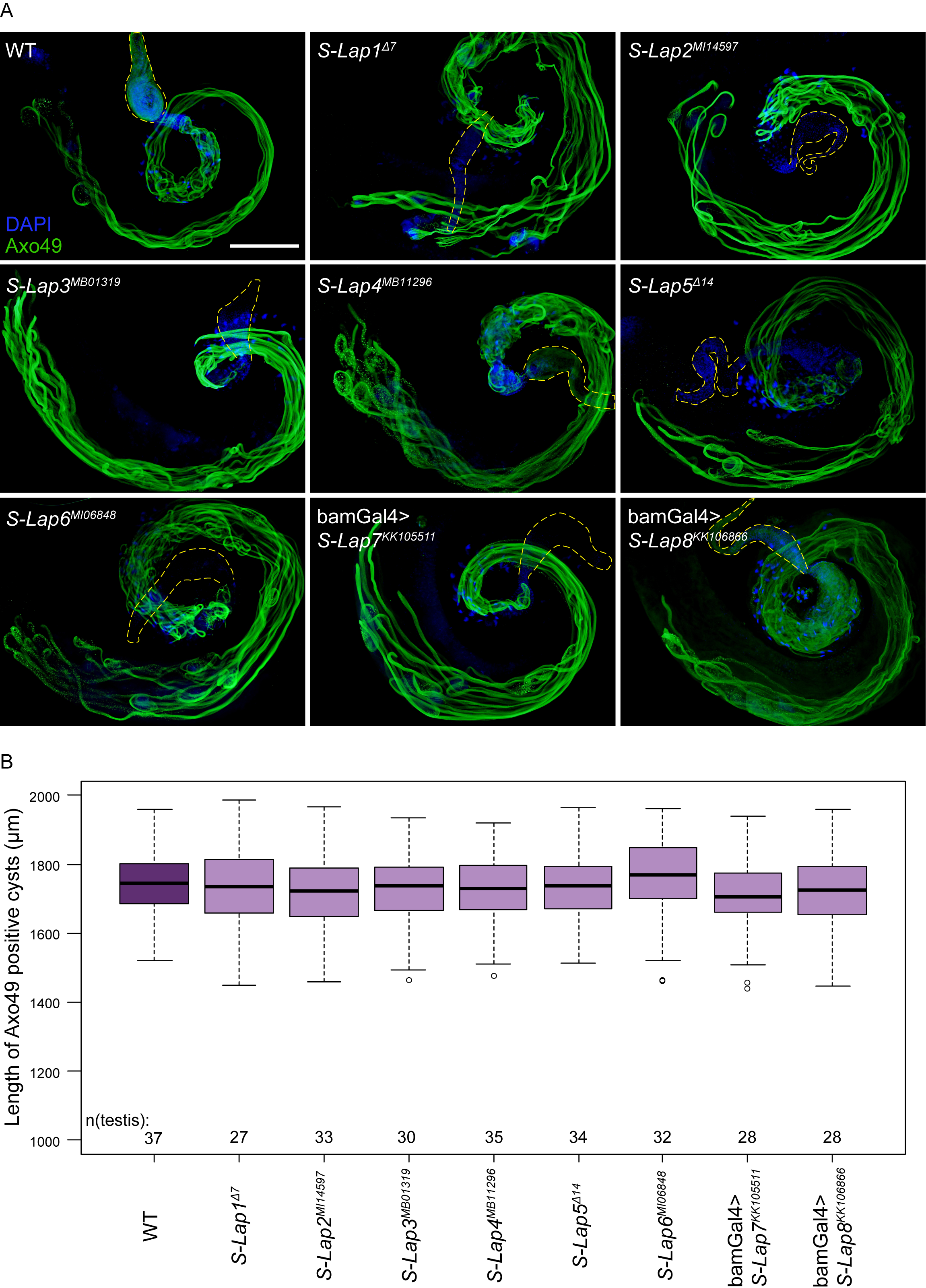

Supplement: S3 Fig — (A) Confocal micrographs of elongated spermatid cysts of wild-type (WT) and S-Lap mutants (S-Lap1Δ7, S-Lap2MI14597, S-Lap3MB01319, S-Lap4MB11296, S-Lap5Δ14, S-Lap6MI06848, bamGal4>S-Lap7KK105511, bamGal4>S-Lap8KK106866) stained with Axo49 tubulin antibody (green) and nuclei with DAPI (blue). Scale bar: 200 μm. (B) The boxplot shows the length of the Axo49 positive cysts in WT and S-Lap mutants. (TIF) [file pgen.1007987.s003.tif]

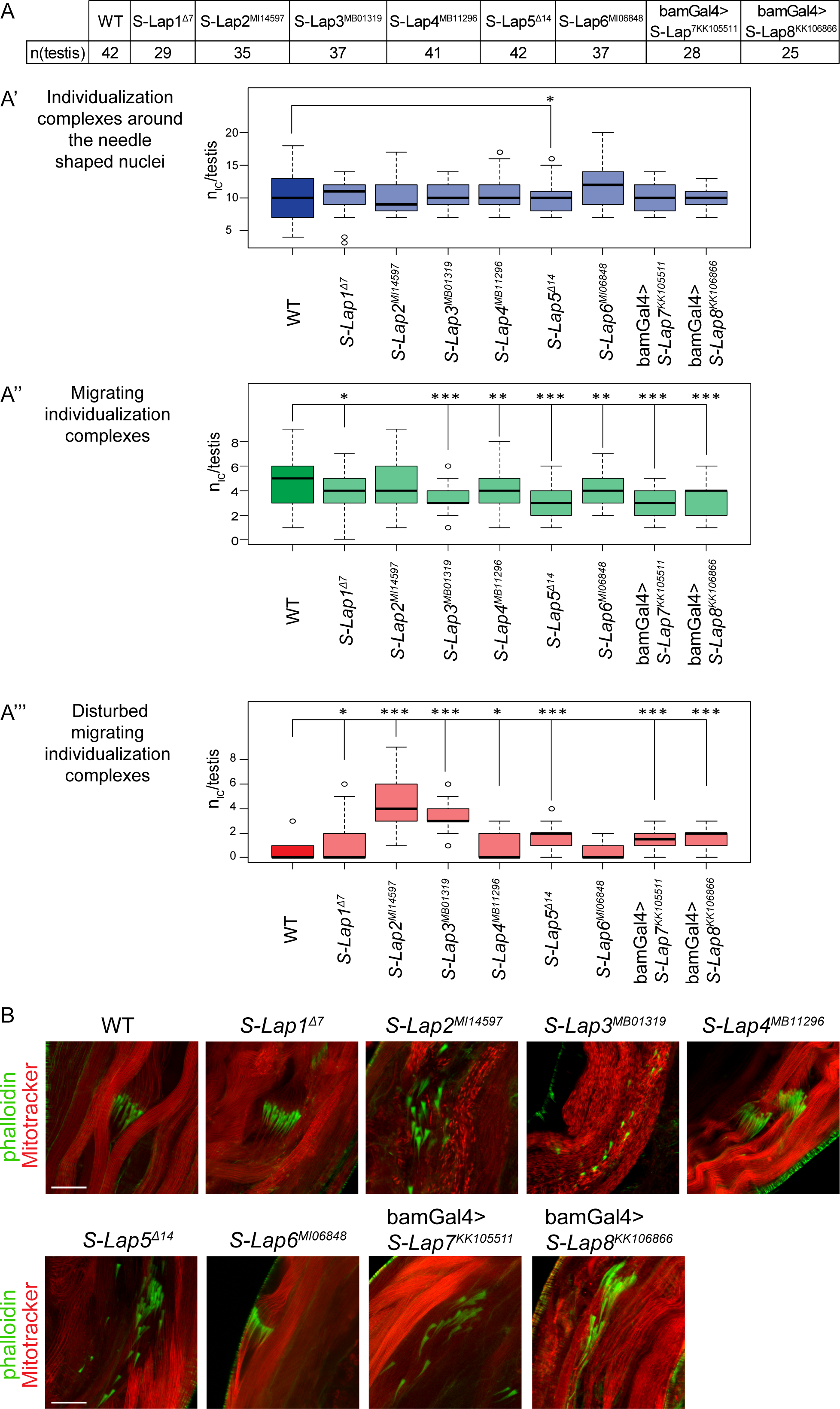

Supplement: S4 Fig — (A) Individualization complexes were counted on phalloidin stained testes samples in S-Lap mutants. n(testis) represents the number of the analyzed testes. (A’) Needle shaped nuclei with individualization complexes, (A”) migrating individualization complexes and (A”‘) disturbed migrating individualization complexes were counted in WT and S-Lap mutants. Statistical significance was determined by Welch two sample t-test. (B) Confocal micrographs of elongated spermatids in wild type (WT)and S-Lap mutants (S-Lap1Δ7, S-Lap2MI14597, S-Lap3MB01319, S-Lap4MB11296, S-Lap5Δ14, S-Lap6MI06848, bamGal4>S-Lap7KK105511, bamGal4>S-Lap8KK106866) show elongated mitochondria stained with Mitotracker (red) and investment cones marked by phalloidin (green) with synchronized and unsynchronized movement. Scale bar: 25 μm. (TIF) [file pgen.1007987.s004.tif]

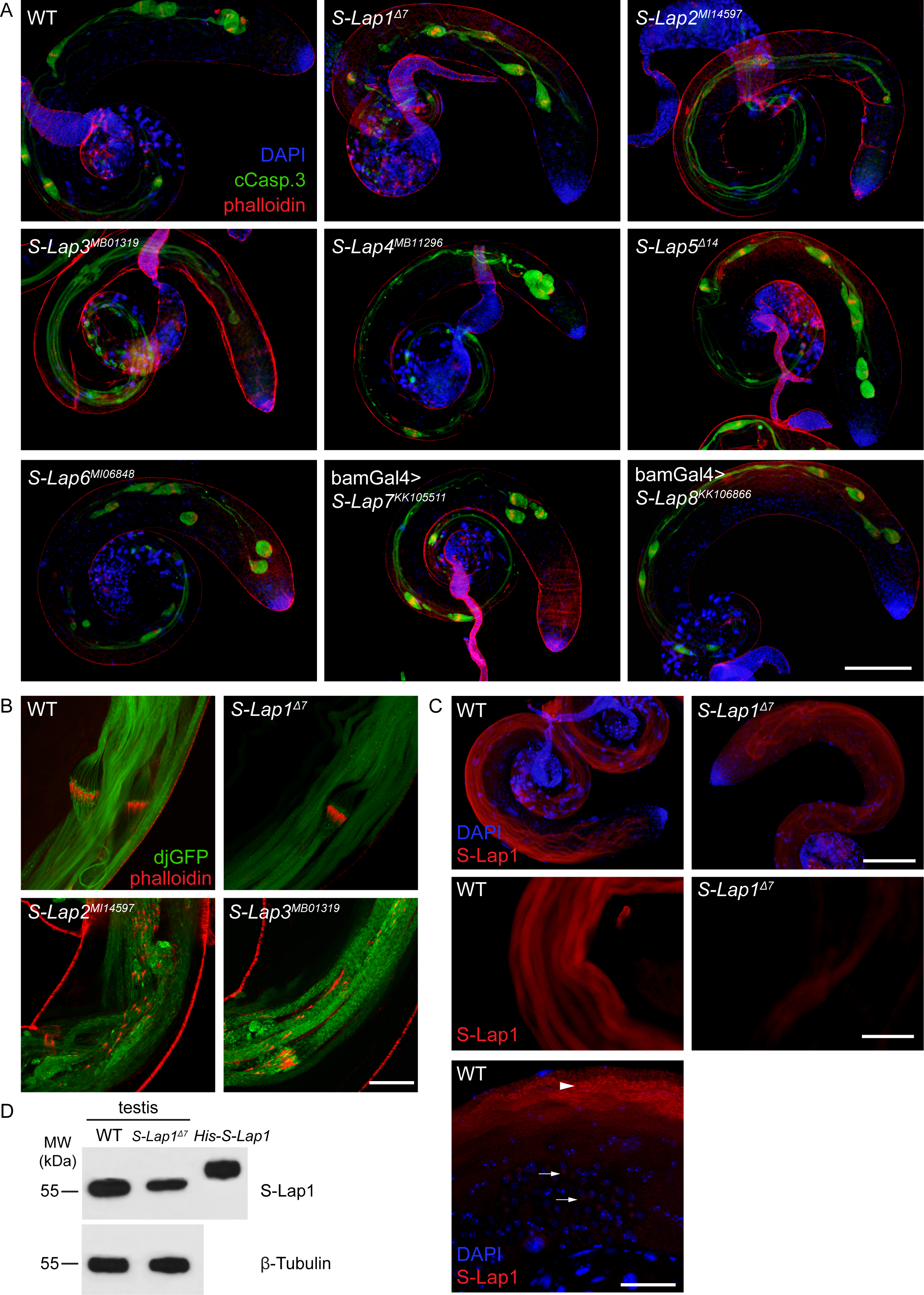

Supplement: S5 Fig — Characterization of the S-Lap1 antibody. (A) Cleaved-Caspase3 (green) is present in the elongated cysts in wild type (WT) and S-Lap mutants (S-Lap1Δ7, S-Lap2MI14597, S-Lap3MB01319, S-Lap4MB11296, S-Lap5Δ14, S-Lap6MI06848, bamGal4>S-Lap7KK105511, bamGal4>S-Lap8KK106866). Individualization complexes were stained with phalloidin (red) and nuclei with DAPI (blue). Scale bar: 200 μm. (B) Investment cones are synchronized and mitochondrial DJ-GFP distribution is smooth, while in the S-Lap1Δ7 mutant investment cones are synchronized and DJ-GFP distribution is slightly dashed. In the S-Lap2MI14597 mutant and the S-Lap3MB01319 mutant, the investment cones are dispersed and DJ-GFP distribution is dashed. Scale bar: 50 μm. (C) S-Lap1 antibody (red) decorates weakly the nebenkern in round spermatids (arrows) and the elongating and the elongated mitochondria (arrowhead) both in WT and S-Lap1 mutants. The signal intensity is reduced in the S-Lap1Δ7 testis by using the same exposure time as in the WT. Nuclei were stained with DAPI (blue). Scale bars: 200 μm and 50 μm. (D) Immunoblot of WT and S-Lap1Δ7 testes using S-Lap1 antibody, where one lane represents 1 testis equivalent total lysate. S-Lap1 antibody recognizes the recombinant His-S-Lap1 protein. Note that the His-tagged unprocessed recombinant protein runs higher (~70 kDa) than the processed and untagged endogenous S-Lap1 (~55 kDa). (TIF) [file pgen.1007987.s005.tif]

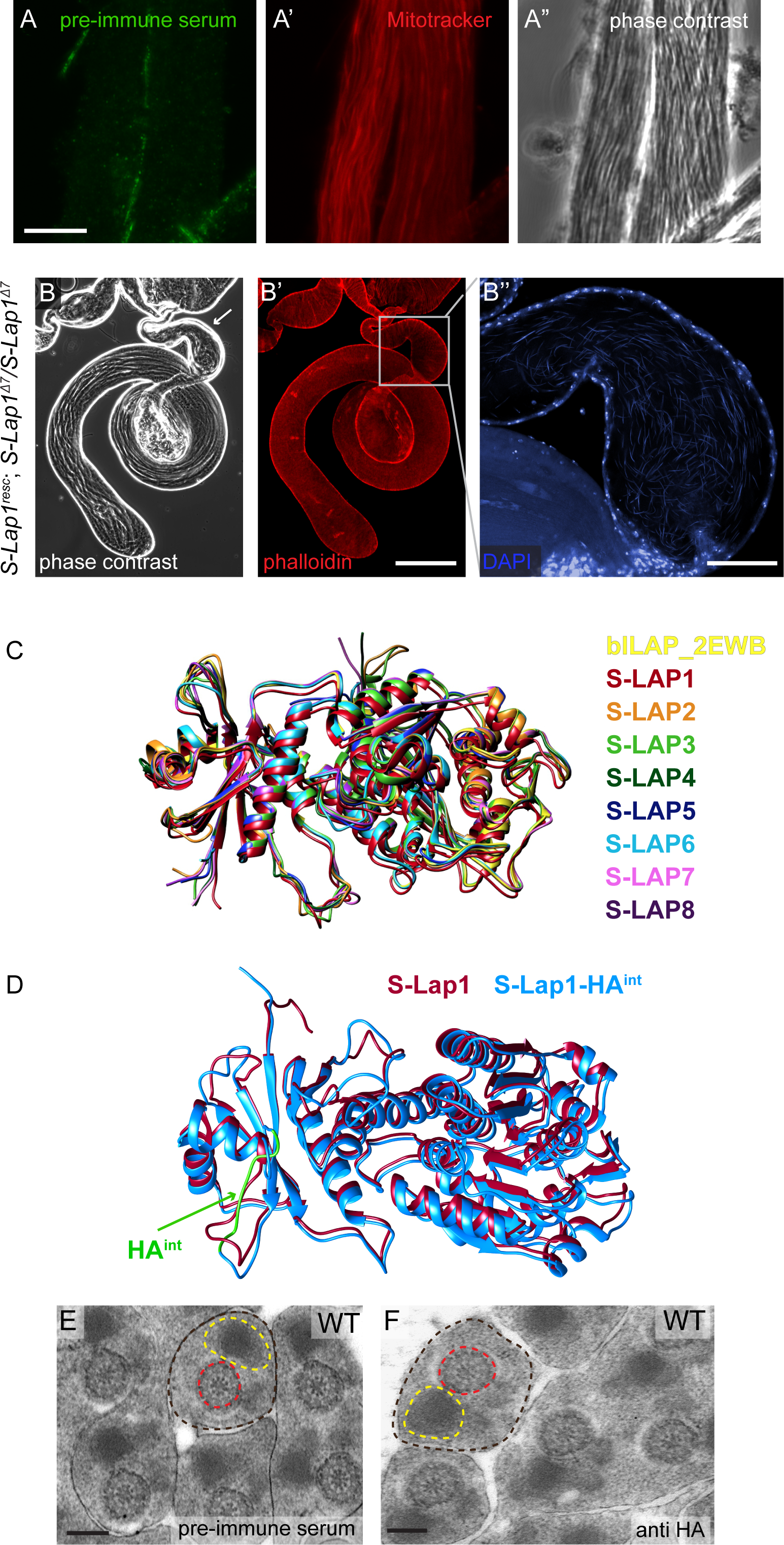

Supplement: S6 Fig — (A, A’, A”) Confocal micrographs of elongated cysts stained with mouse pre-immune serum of S-Lap1 antibody (green), and Mitotracker (red) in squash preparation of wild-type testis. Scale bar: 10 μm. (B) Phase contrast and fluorescent images (B’, B”) of the testis of the S-Lap1Δ7 mutant expressing the S-Lap1 genomic rescue construct (S-Lap1resc), stained with phalloidin (red) and the nuclei with DAPI (blue). The seminal vesicle contains individualized sperms (arrow on B, grey square on B’ and in larger magnification on B”). Scale bars: 200 μm (B, B’) 50 μm (B”). (C) Three-dimensional models of S-Lap proteins compared to the bovine leucine aminopeptidase protein model 2EWB. (D) Three-dimensional model alignment of S-Lap1 (red) and S-Lap1-HAint (blue) and the position of the internal 1xHA tag (green). (E, F) Cryo cross-sections of wild-type spermatids immuno-labeled with (E) pre-immune serum of S-Lap1 antibody and anti-HA antibody (F) do not show specific labeling after developing with nanogold particles. Brown dashed line borders one elongated spermatid with axoneme (red dashed line) and with major mitochondrial derivative (yellow dashed line) inside. Scale bars: 0.2 μm. (TIF) [file pgen.1007987.s006.tif]

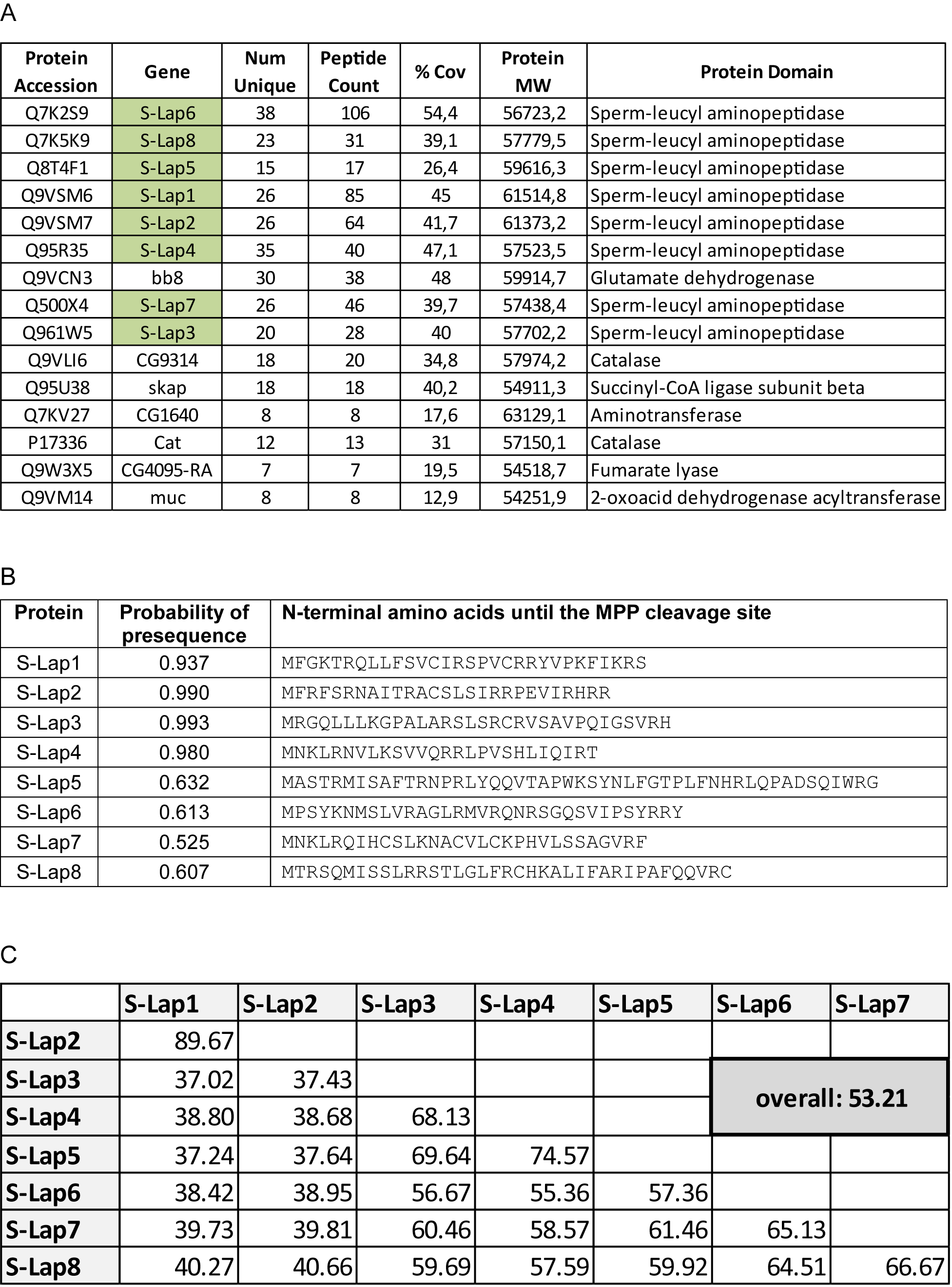

Supplement: S7 Fig — (A) Analysis of protein accumulation in the 50-60 kDa molecular weight region of SDS-resistant pellet fraction of sperm extract by mass spectrometry. (B) S-Lap proteins contain an N-terminal mitochondrial target sequence with high probability. The MPP (mitochondrial processing peptidase) cleavage sites were predicted using the online software MitoFates. (C) Sequence similarity between S-Lap proteins. Alignment analysis was made by Clustal Omega software. (TIF) [file pgen.1007987.s007.tif]

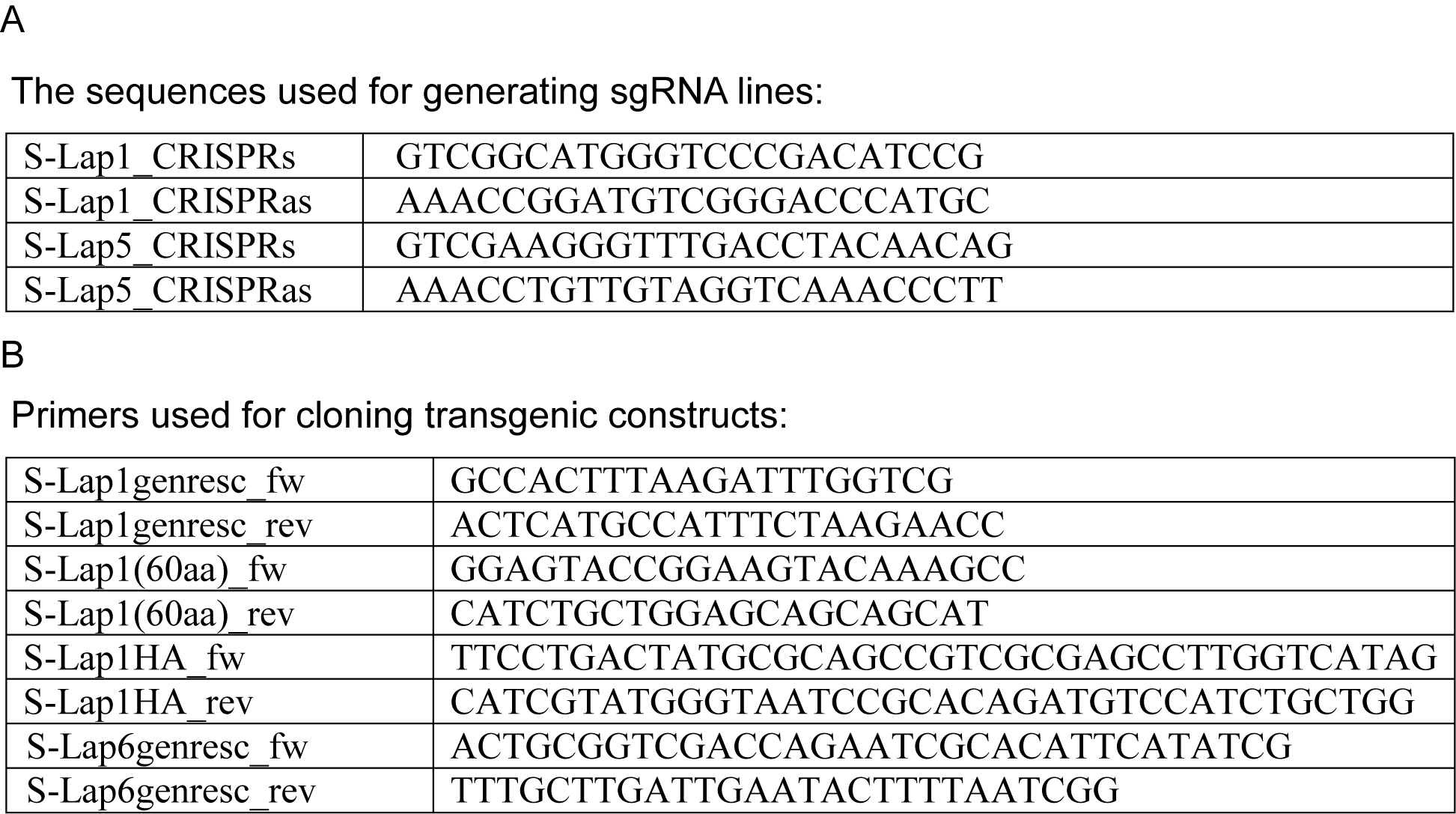

Supplement: S8 Fig — (A) The sequences used for generating sgRNA lines and (B) the primers used for cloning transgenic constructs. (TIF) [file pgen.1007987.s008.tif]
